# Author Correction: The shaky foundations of simulating single-cell RNA sequencing data

**DOI:** 10.1186/s13059-024-03329-0

**Published:** 2024-07-05

**Authors:** Helena L. Crowell, Sarah X. Morillo Leonardo, Charlotte Soneson, Mark D. Robinson

**Affiliations:** 1 Department of Molecular Life Sciences, University of Zurich, Zurich, Switzerland; 2 SIB Swiss Institute of Bioinformatics, University of Zurich, Zurich, Switzerland; 3 ETH Zurich, Zurich, Switzerland; 4 Present address: Friedrich Miescher Institute for Biomedical Research and SIB Swiss Institute of Bioinformatics, Basel, Switzerland

**Correction: Genome Biol 24, 62 (2023)**

**https://doi.org/10.1186/s13059-023-02904-1**

Following publication of the original article [1], it was pointed out that the legend to Table 1 did not match the content of the table.

The incorrect Table 1 is as follows:

**Table 1:** Overview of scRNA-seq simulators compared in this study. Methods are ordered alphabetically and annotated according to their (in)ability to accommodate multiple batches and/or clusters, support for parallelization (parameter estimation and data simulation, respectively), software availability, and publication year. The right-most column catalogues neutral benchmark studies where each simulator was used. (✓ = yes, ✘ = no, (✓) = yes, but based on user input parameters, i.e., no support for parameter estimation, *requires random splitting of cells into two groups, †/‡ = internal/prior resampling from empirical parameter distribution, ∘ = no separate estimation step)

	**Batches**	**Clusters**	**Type(s)**	**Cell #**	**Parallelization**	**Availability**	**Year**	**Model**
BASiCS [37]	✓	✘	b	✘	✓✘	R/Bioc	2015	NB
ESCO [38]	✓	✓	n,b,k	✓	✓✓	R/GitHub	2020	Gamma-Poisson
hierarchicell [39]	✓	✘	n,b	✓	✘✘	R/GitHub	2021	NB
muscat [40]	✓	✓	n,b,k	(✓)†	✘✘	R/Bioc	2020	NB
POWSC [41]	✘	✓	n,k	(✓)†	✘✘	R/Bioc	2020	zero-inflated, log-normal Poisson mixture
powsimR [42]	✘	(✓)	n*	(✓)†	✓✓	R/GitHub	2017	NB
scDD [43]	✘	✘	n*	✓	✓✓	R/Bioc	2016	Bayesian NB mixture model
scDesign [44]	✘	(✓)	n	✓	∘✓	R/GitHub	2019	Gamma-Normal mixture model
scDesign2 [45]	✘	✓	n,k	✓	✓✘	R/GitHub	2020	(zero-inflated) Poisson or NB + Gaussian copula for gene–gene correlations
SCRIP [46]	✓	✓	n,b,k	✓	✘✘	R/GitHub	2020	(Beta-)Gamma-Poisson
SPARSim [47]	✓	✘	n,b	(✓)‡	✘✘	R/GitLab	2020	Gamma-multivariate hypergeometric
splatter [15] (Splat model)	(✓)	(✓)	n	✓	✘✘	R/Bioc	2017	Gamma-Poisson
SPsimSeq [16]	✓	✘	n,b	✓	∘✘	R/Bioc	2020	log-linear model-based density estimation + Gaussian copula for gene–gene correlations
SymSim [48]	✓	✘	n,b	✓	✘✘	R/GitHub	2019	kinetic model using MCMC
ZINB-WaVE [49]	✓	✓	n,b,k	✘	✘✘	R/Bioc	2018	zero-inflated NB
zingeR [50]	✘	✘	n	(✓)†‡	✘✘	R/GitHub	2017	zero-inflated NB

The corrected Table 1 is as follows:

**Table 1:** Overview of scRNA-seq simulators compared in this study. Methods are ordered alphabetically and annotated according to their (in)ability to accommodate multiple batches and/or clusters, support for parallelization (parameter estimation and data simulation, respectively), software availability, and publication year. ‘Type(s)’ column specifies which type of simulations can be produced (n: “singular” references: single batch or cluster; b: multiple batches; k: multiple clusters). ‘Cell #’ refers to whether the number of cells can be varied. Symbols: ✓ = yes, ✘ = no, (✓) = yes, but based on user input parameters, i.e., no support for parameter estimation, *requires random splitting of cells into two groups, †/‡ = internal/prior resampling from empirical parameter distribution, ∘ = no separate estimation step)

	**Batches**	**Clusters**	**Type(s)**	**Cell #**	**Parallelization**	**Availability**	**Year**	**Model**
BASiCS [37]	✓	✘	b	✘	✓✘	R/Bioc	2015	NB
ESCO [38]	✓	✓	n,b,k	✓	✓✓	R/GitHub	2020	Gamma-Poisson
hierarchicell [39]	✓	✘	n,b	✓	✘✘	R/GitHub	2021	NB
muscat [40]	✓	✓	n,b,k	(✓)†	✘✘	R/Bioc	2020	NB
POWSC [41]	✘	✓	n,k	(✓)†	✘✘	R/Bioc	2020	zero-inflated, log-normal Poisson mixture
powsimR [42]	✘	(✓)	n*	(✓)†	✓✓	R/GitHub	2017	NB
scDD [43]	✘	✘	n*	✓	✓✓	R/Bioc	2016	Bayesian NB mixture model
scDesign [44]	✘	(✓)	n	✓	∘✓	R/GitHub	2019	Gamma-Normal mixture model
scDesign2 [45]	✘	✓	n,k	✓	✓✘	R/GitHub	2020	(zero-inflated) Poisson or NB + Gaussian copula for gene–gene correlations
SCRIP [46]	✓	✓	n,b,k	✓	✘✘	R/GitHub	2020	(Beta-)Gamma-Poisson
SPARSim [47]	✓	✘	n,b	(✓)‡	✘✘	R/GitLab	2020	Gamma-multivariate hypergeometric
splatter [15] (Splat model)	(✓)	(✓)	n	✓	✘✘	R/Bioc	2017	Gamma-Poisson
SPsimSeq [16]	✓	✘	n,b	✓	∘✘	R/Bioc	2020	log-linear model-based density estimation + Gaussian copula for gene–gene correlations
SymSim [48]	✓	✘	n,b	✓	✘✘	R/GitHub	2019	kinetic model using MCMC
ZINB-WaVE [49]	✓	✓	n,b,k	✘	✘✘	R/Bioc	2018	zero-inflated NB
zingeR [50]	✘	✘	n	(✓)†‡	✘✘	R/GitHub	2017	zero-inflated NB

The linked citations and the hyperlinks to the availability of data (in table 1) can be found in the original article. The original article [1] has been corrected.
