# Supplementary material for: The shaky foundations of simulating single-cell RNA sequencing data
Source: Genome Biol. 2023 Mar 29;24:62. doi: 10.1186/s13059-023-02904-1 (PMC10061781; doi:10.1186/s13059-023-02904-1)
Supplement: Supplementary file 2 — Additional file 2: Figures S1-3 (t-SNEs of reference datasets), Figs. S4-10 (comparison of 1/2D test statistics), Figs. S11-13 (runtime and memory usage), Figs. S14-16. (integration results), Figs. S17-24 (t-SNEs of integrated datasets), Figs. S25-28 (meta-analysis of summaries). [file 13059_2023_2904_MOESM2_ESM.pdf]

# Supplementary figures:

# The shaky foundations of simulating single-cell RNA sequencing data

Helena L. Crowell[1,2], Sarah X. Morillo Leonardo[3], Charlotte Soneson[1,2,4] & Mark D. Robinson[1,2]

[1] *Department of Molecular Life Sciences, University of Zurich, Zurich, Switzerland*

[2] *SIB Swiss Institute of Bioinformatics, University of Zurich, Switzerland*

[3] *ETH Zurich, Zurich, Switzerland*

[4] *Current address: Friedrich Miescher Institute for Biomedical Research and SIB Swiss Institute of Bioinformatics, Basel, Switzerland*

## Contents

**1 Dimension reduction** **1**

**2 Evaluation statistics** **4**

2.1 One-dimensional . . . . . . . . . . . . . . . . . . . . . . . . . . . . . . . . . . . . . . 5

2.2 Two-dimensional . . . . . . . . . . . . . . . . . . . . . . . . . . . . . . . . . . . . . . 8

**3 Scalability** **11**

**4 Downstream** **14**

4.1 Integration . . . . . . . . . . . . . . . . . . . . . . . . . . . . . . . . . . . . . . . . . 14

4.2 Summaries . . . . . . . . . . . . . . . . . . . . . . . . . . . . . . . . . . . . . . . . . 21

# 1 Dimension reduction

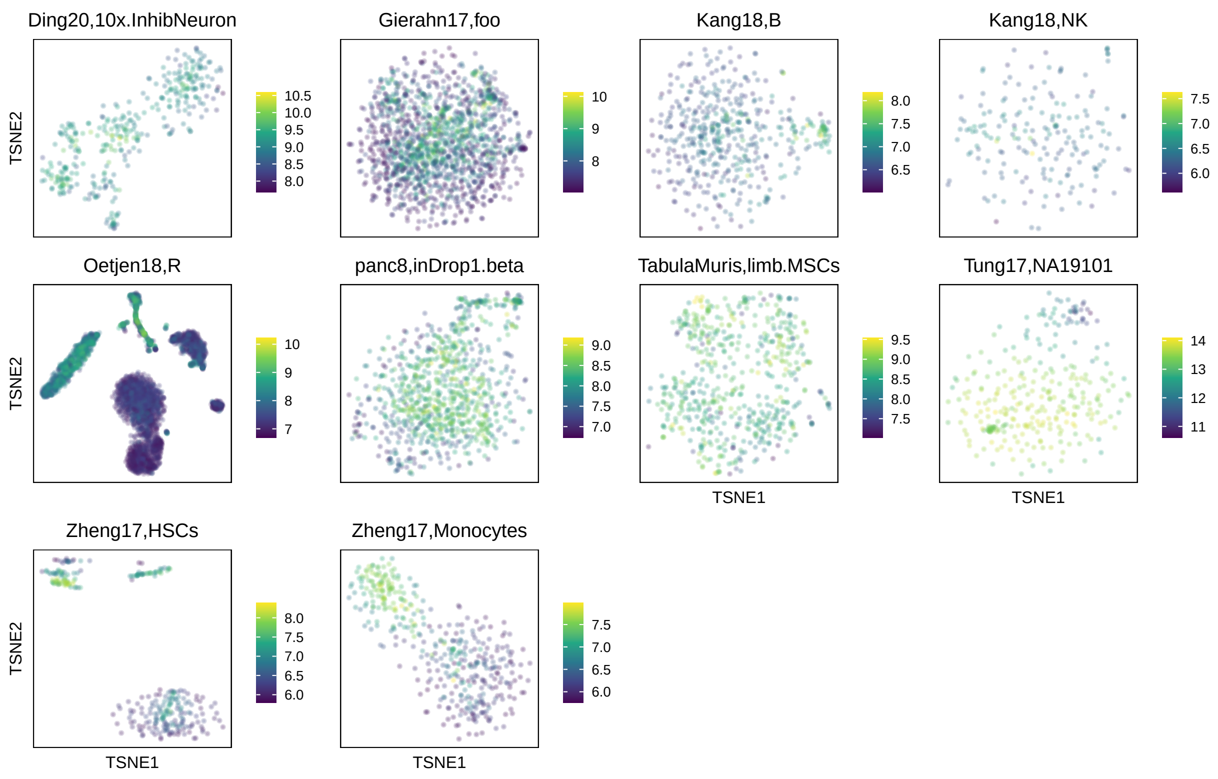


**Fig. S1:** t-SNE plots of type $n$ datasets. Cells (points) are colored by log1p-transformed library size (total counts). Plot titles correspond to comma-separated reference and subset identifiers.

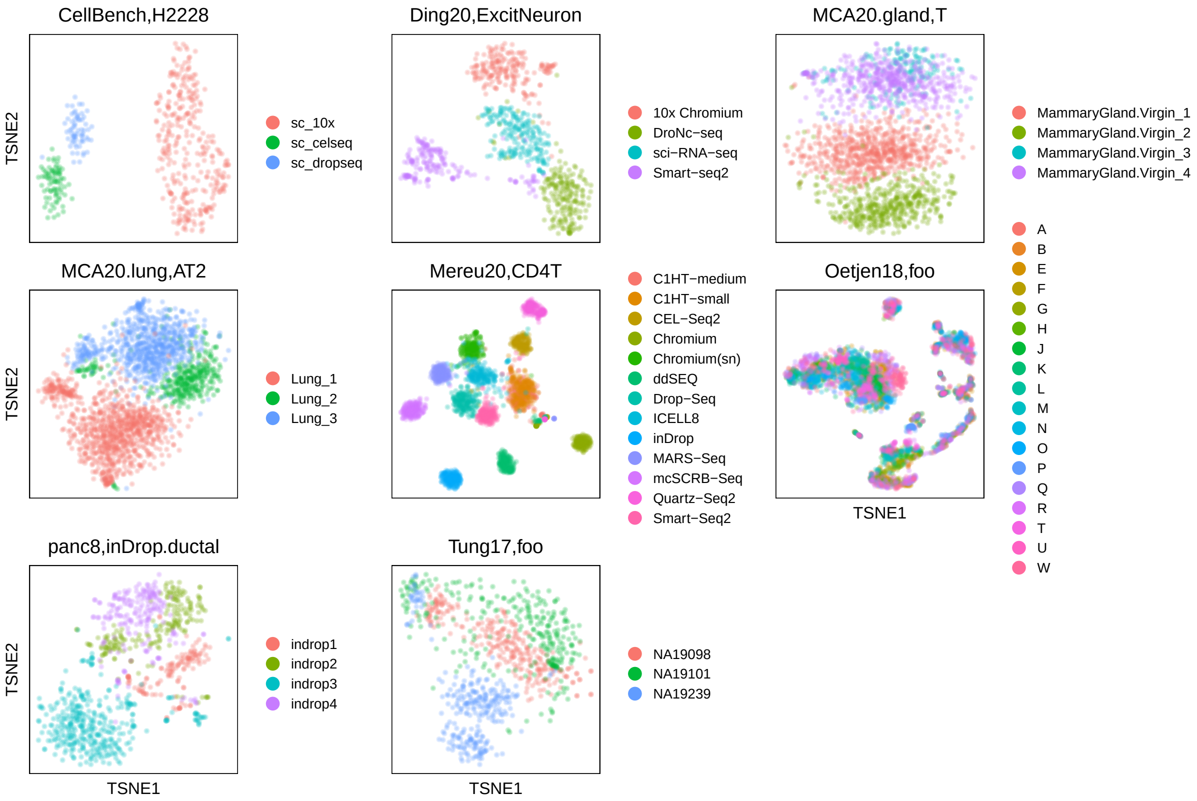


**Fig. S2:** t-SNE plots of type $b$ datasets. Cells (points) are colored by batch (biological or technical replicate). Plot titles correspond to comma-separated reference and subset identifiers.

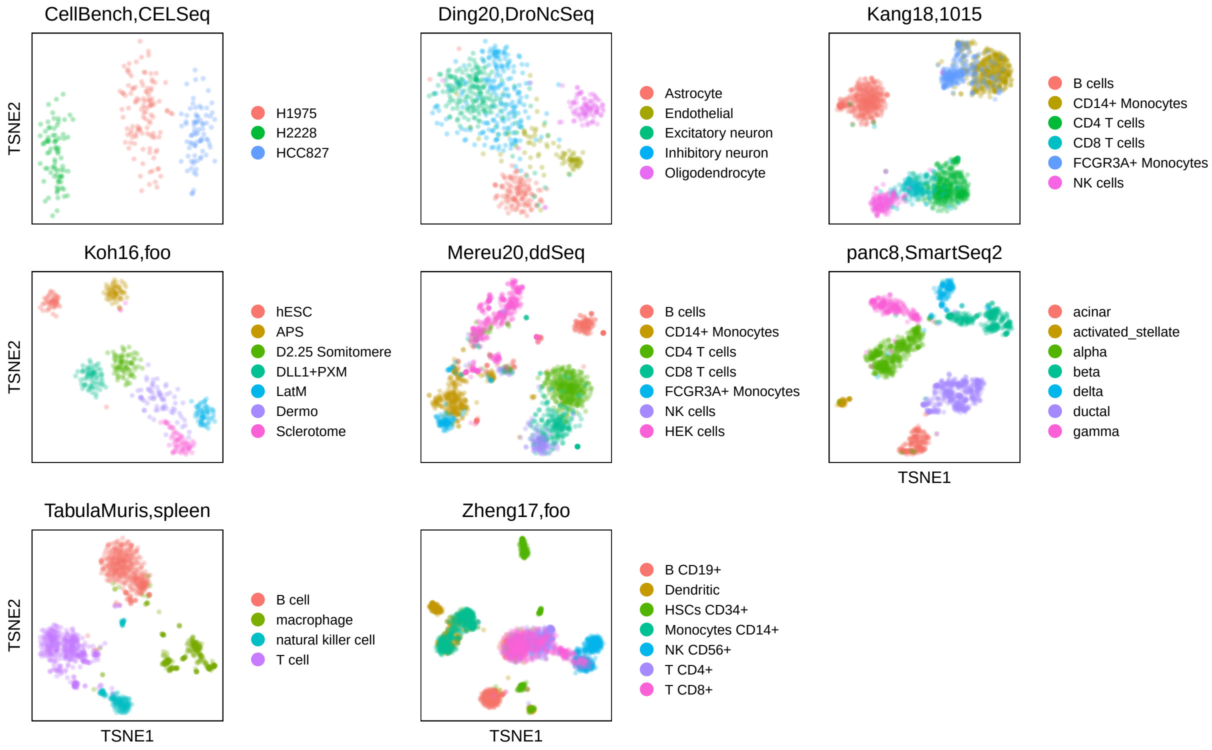


**Fig. S3:** t-SNE plots of type $k$ datasets. Cells (points) are colored by cluster (cell subpopulation). Plot titles correspond to comma-separated reference and subset identifiers.

## 2 Evaluation statistics

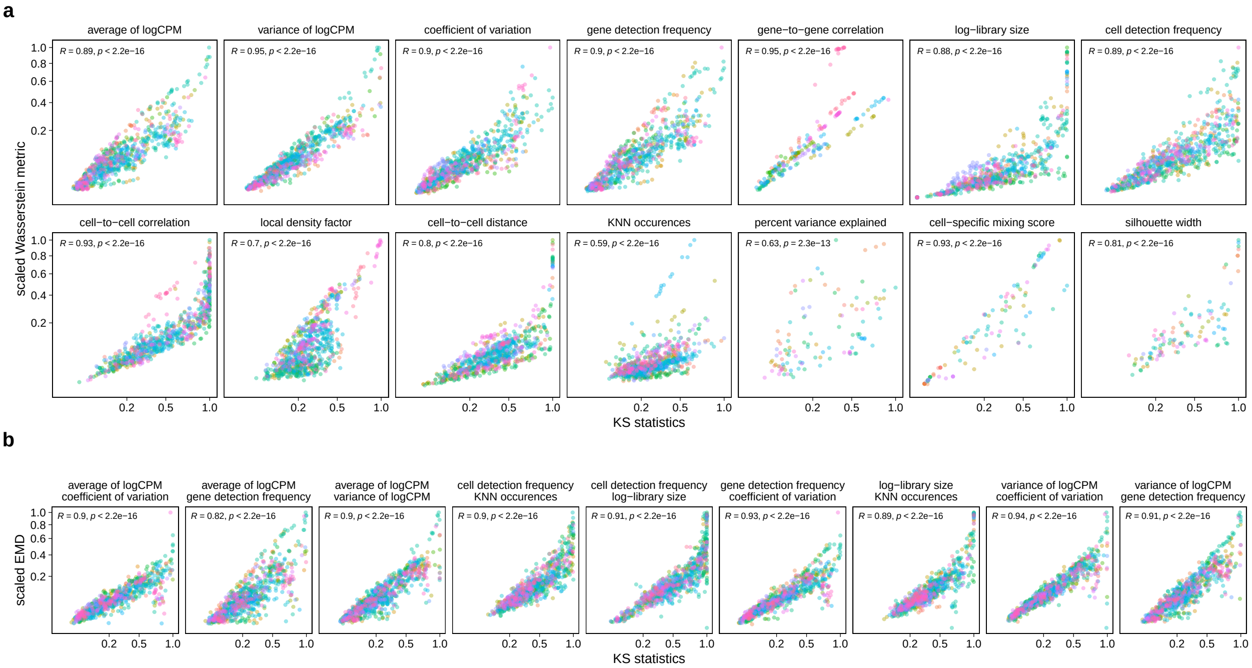


**Fig. S4:** Comparison of (a) one- and (b) two-dimensional test statistics. Each point corresponds to test statistics obtained for a given metric, method, dataset, and (if applicable) group (i.e., batch or cluster); points are colored by dataset. For comparability, Wasserstein metric and EMD values (y-axes) are scaled between 0 and 1 for each metric (panel). For datasets other than type $n$, only batch- and cluster-level (not global) results are included. Annotations represent Pearson correlation coefficients ($R$) and corresponding p-values ($p$).

## 2.1 One-dimensional

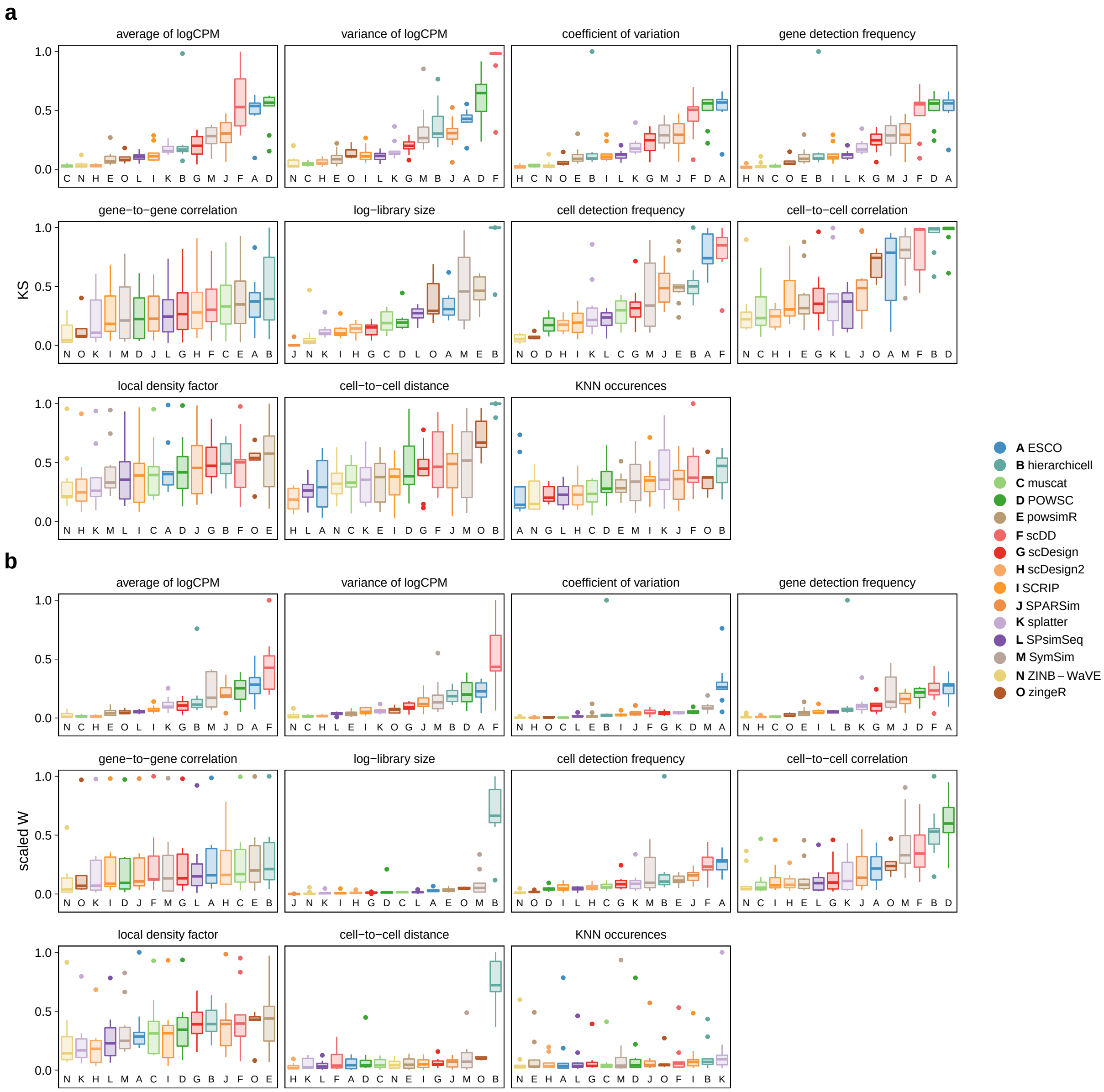


**Fig. S5:** Comparison of KS statistics (a) and Wasserstein metrics (b), type $n$. For each metric (panel), methods (x-axis) are ordered according to their average.

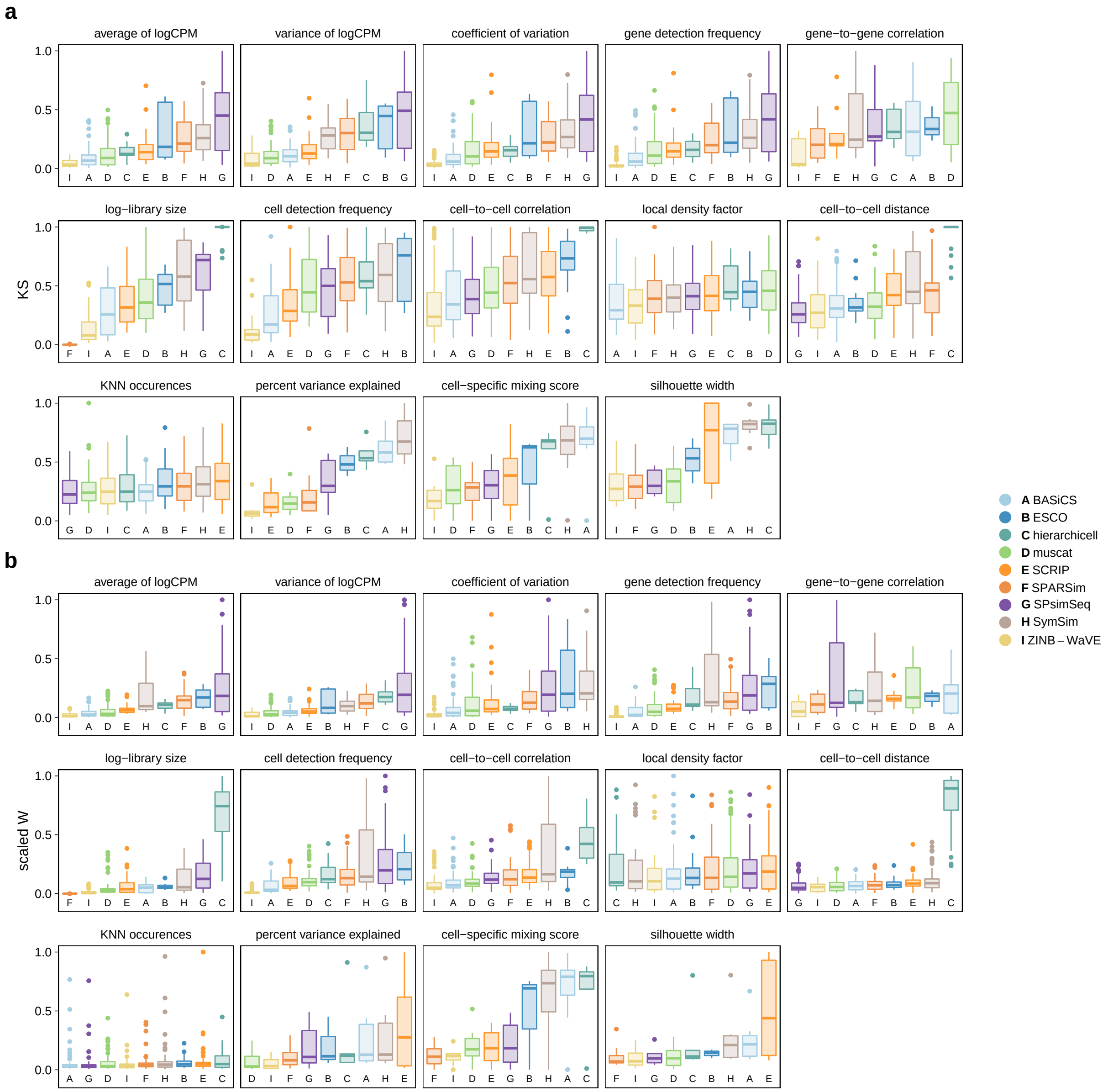


**Fig. S6:** Comparison of KS statistics (a) and Wasserstein metrics (b), type $n$. For each metric (panel), methods (x-axis) are ordered according to their average.

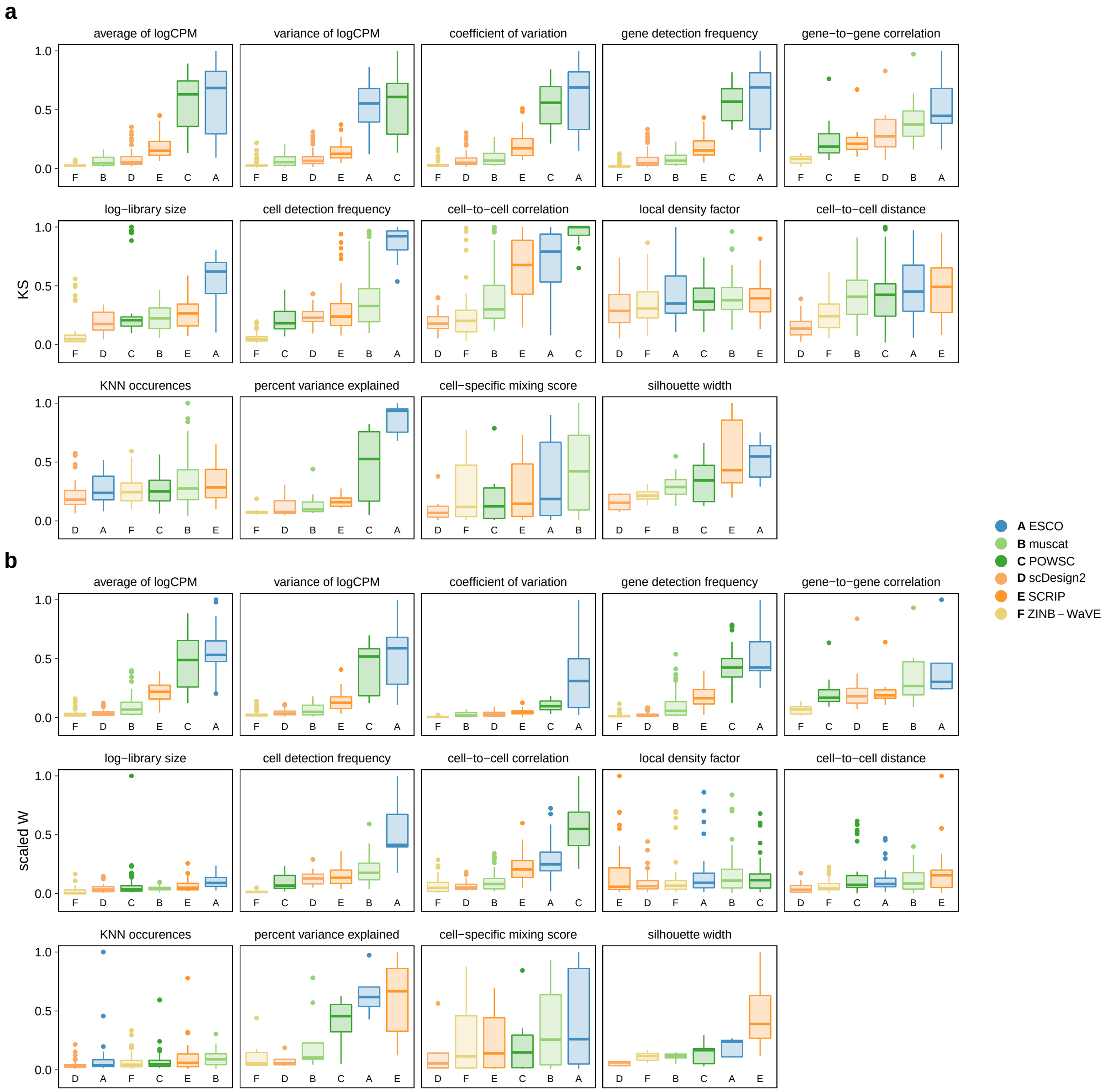


**Fig. S7:** Comparison of KS statistics (a) and Wasserstein metrics (b), type *b*. For each metric (panel), methods (x-axis) are ordered according to their average.

## 2.2 Two-dimensional

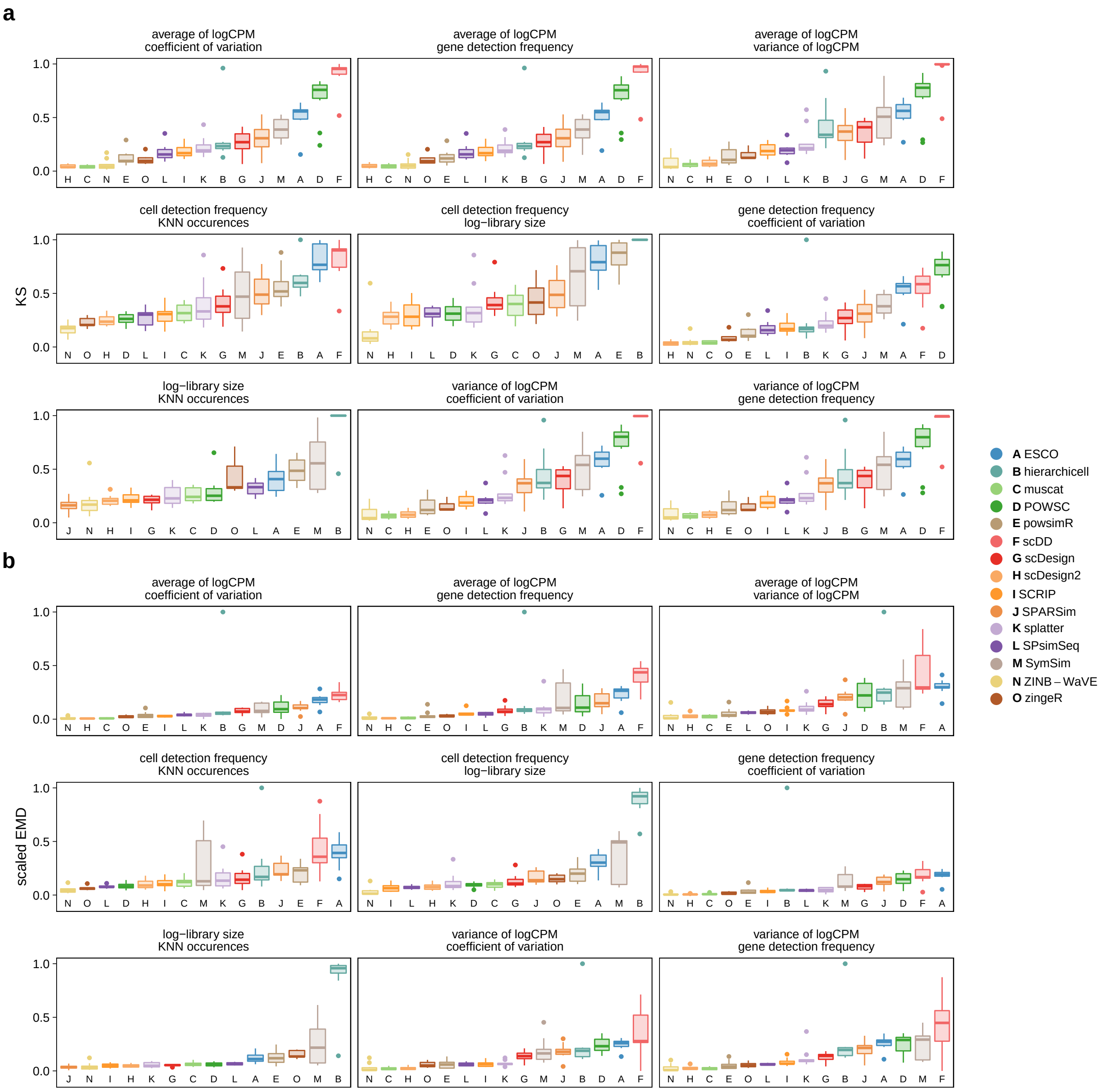


**Fig. S8:** Comparison of KS statistics (a) and EMDs (b), type *n*. For each pair of metrics (panel), methods (x-axis) are ordered according to their average.

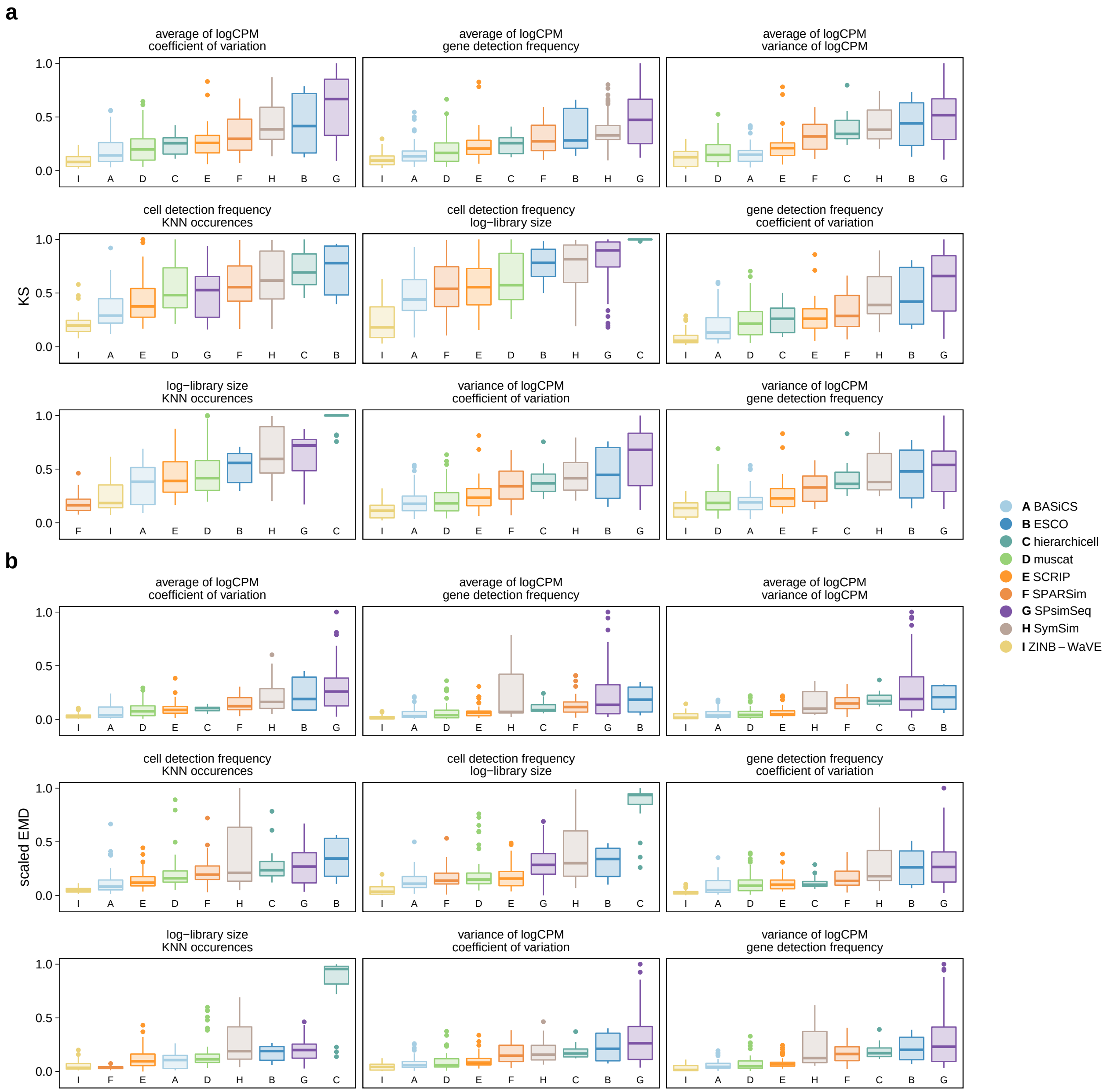


**Fig. S9:** Comparison of KS statistics (a) and EMDs (b), type *b*. For each pair of metrics (panel), methods (x-axis) are ordered according to their average.

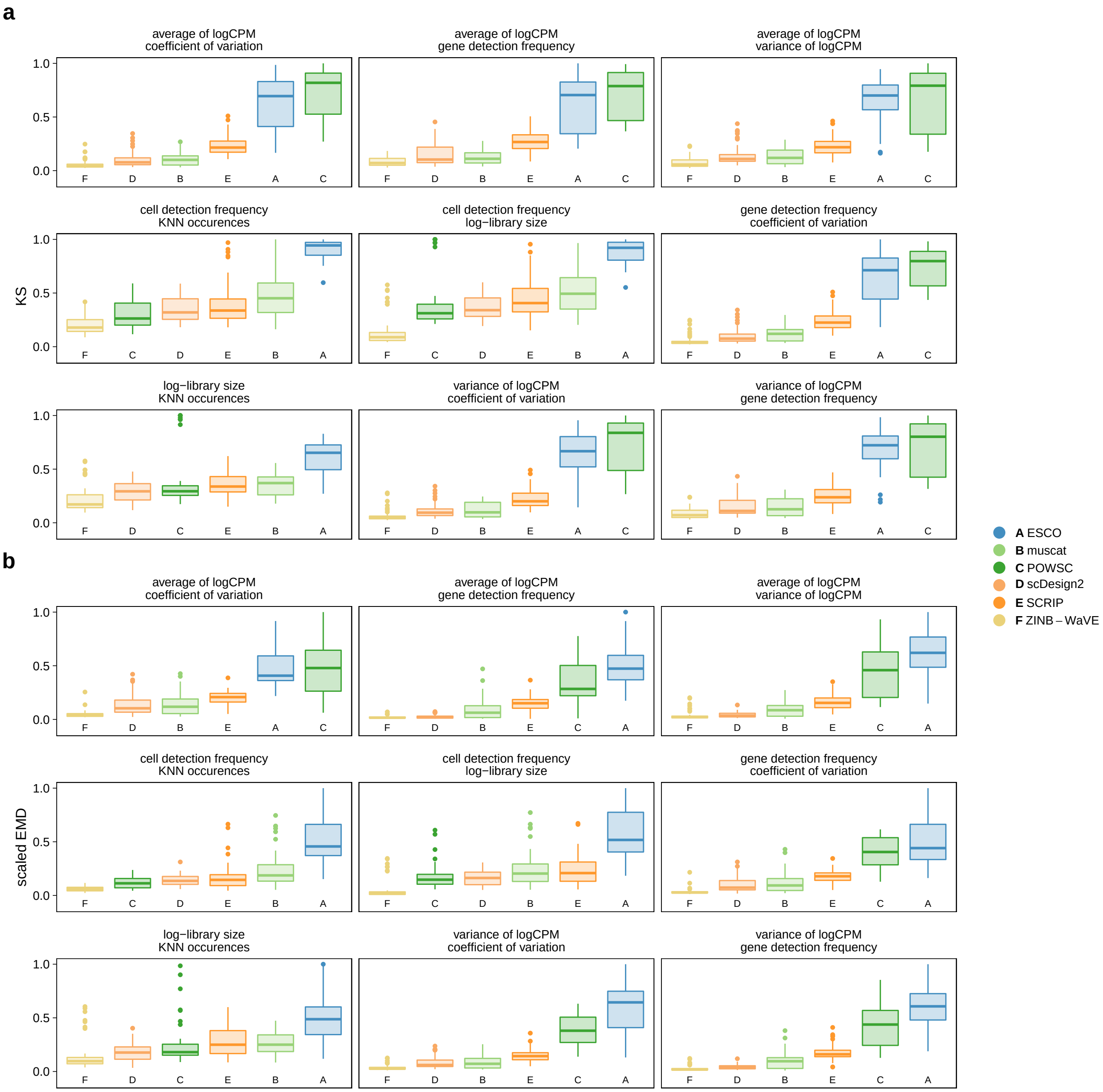


**Fig. S10:** Comparison of KS statistics (a) and EMDs (b), type $k$. For each pair of metrics (panel), methods (x-axis) are ordered according to their average.

# 3 Scalability

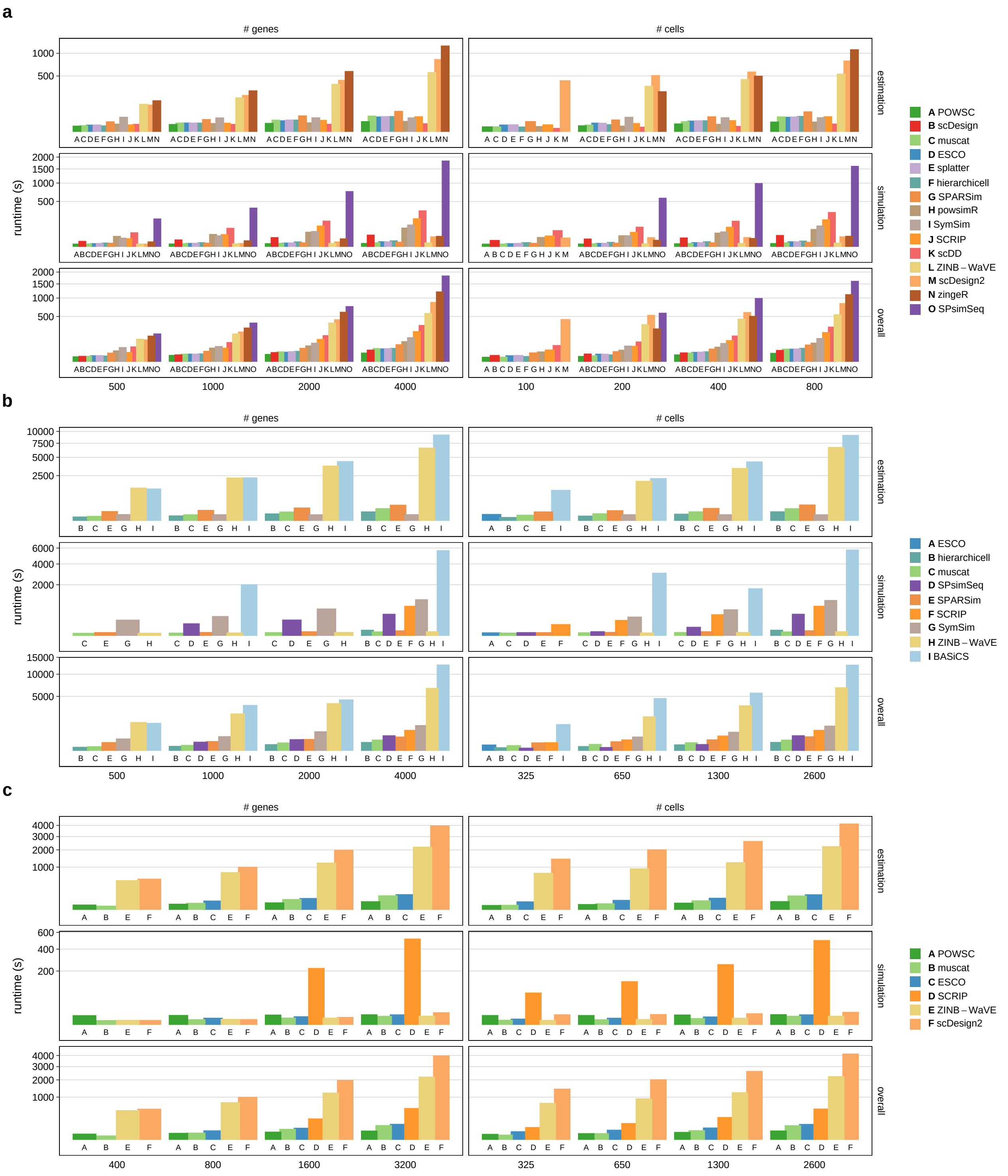


**Fig. S11:** Comparison of parameter estimation (optional), data simulation, and overall runtimes; stratified by type: (a) $n$, (b) $b$, and (c) $k$. Bars correspond to runtimes in seconds (s) averaged across 5 replicates per number of genes and cells, respectively. For each replicate, the number of genes (cells) was fixed when downsampling cells (genes). Methods are ordered by their average overall runtime across all gene and cell subsets.

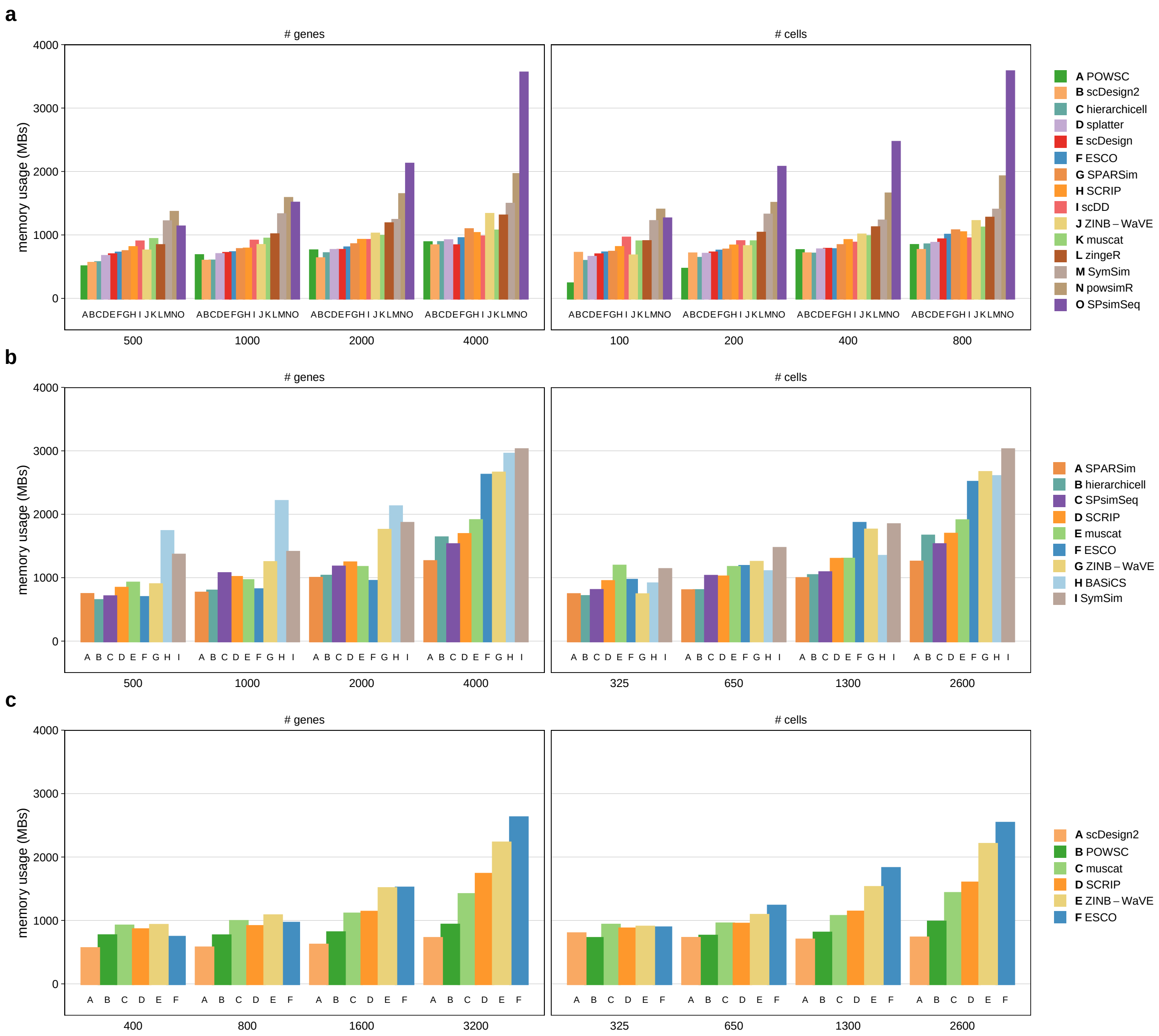


**Fig. S12:** Comparison of memory usage; stratified by type: (a) $n$, (b) $b$, and (c) $k$. Bars correspond to Resident Set Size (RSS) in megabytes (MBs) averaged across 5 replicates per number of genes and cells, respectively. For each replicate, the number of genes (cells) was fixed when downsampling cells (genes). Methods are ordered by their average memory usage across all gene and cell subsets.

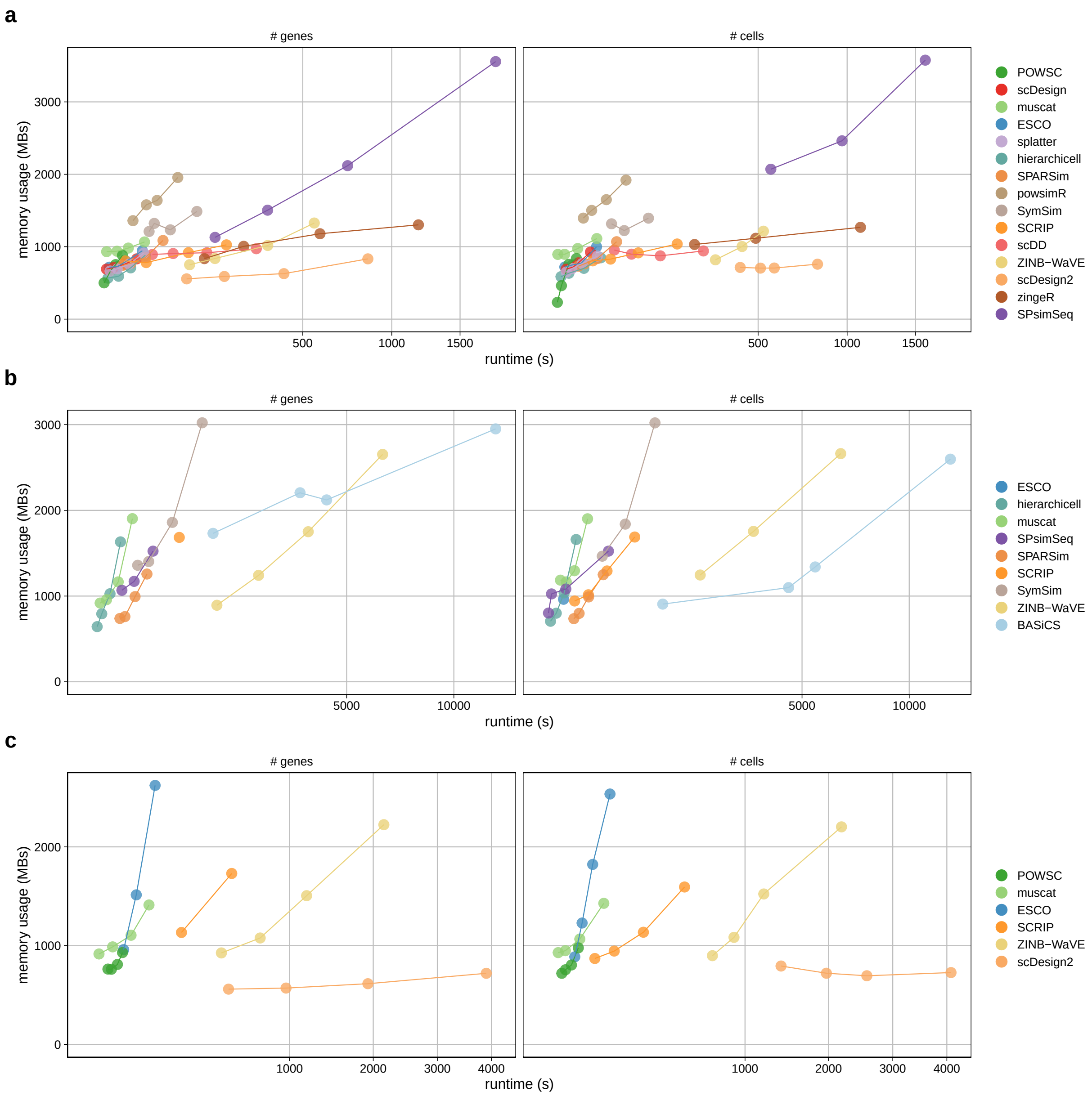


**Fig. S13:** Runtime vs. memory usage; stratified by type: (a) $n$, (b) $b$, and (c) $k$. Each point corresponds to overall (i.e., parameter estimation and simulation) runtime (s) and Resident Set Size (RSS) in megabytes (MBs) averaged across 5 replicates for a given gene and cell subset, respectively. For each replicate, the number of genes (cells) was fixed when downsampling cells (genes).

# 4 Downstream

## 4.1 Integration

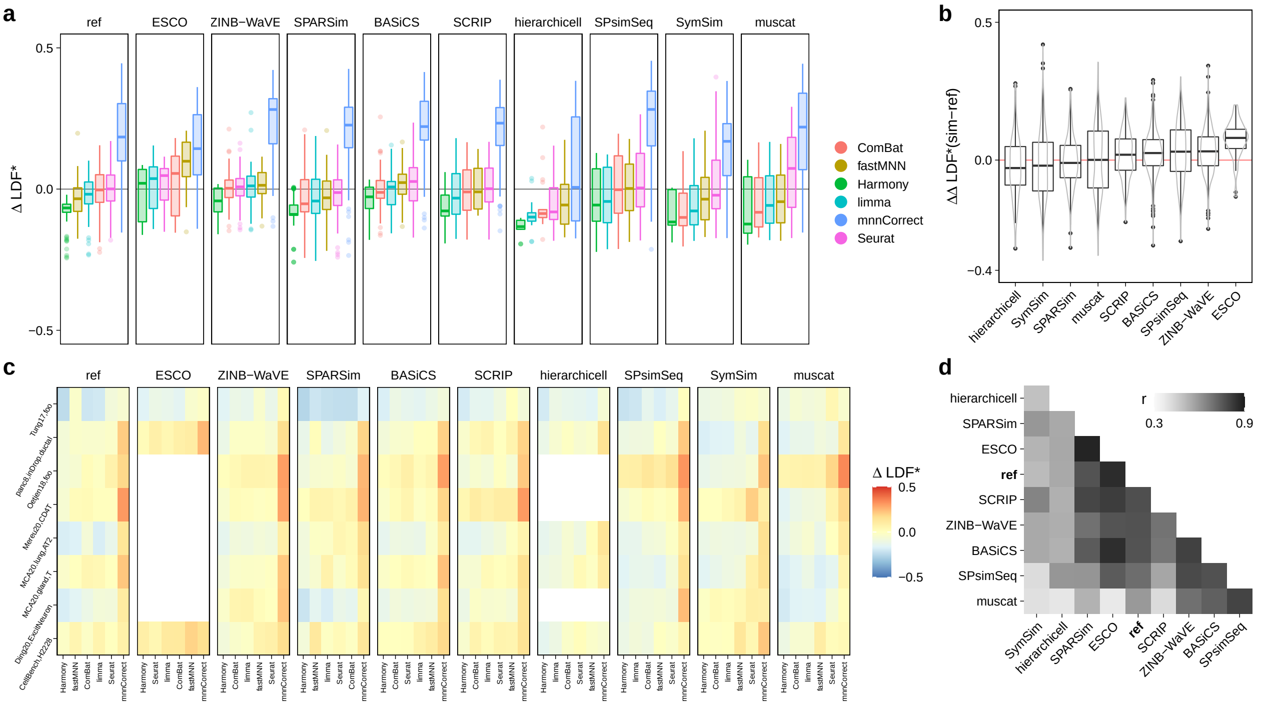


**Fig. S14:** Comparison of integration results across (experimental) reference and (synthetic) simulated data. (a) Boxplot of batch-level difference in local density factors (ΔLDF) across all type $b$ references, simulation and integration methods. (b) Boxplot of difference (Δ) in batch-level ΔLDF obtained from *ref*erence and *sim*ulated data. (c) Heatmap of dataset-level ΔLDF across integration methods (columns) and datasets (rows), stratified by simulator (panels). (d) Heatmap of Spearman's rank correlation ($\rho$) between ΔLDF across datasets and integration methods.

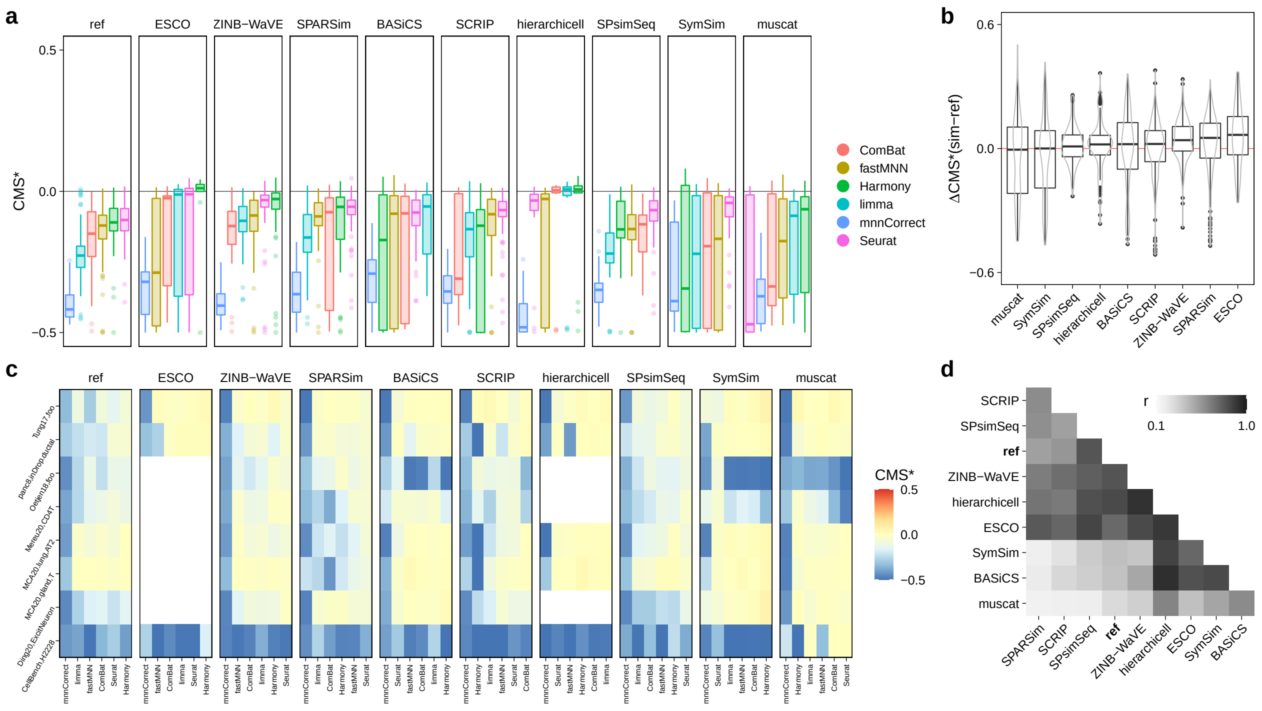


**Fig. S15:** Comparison of integration results across (experimental) reference and (synthetic) simulated data. (a) Boxplot of batch-level cell-specific mixing scores (CMS) across all type $b$ references, simulation and integration methods. (b) Boxplot of difference ($\Delta$) in batch-level CMS obtained from *ref*erence and *sim*ulated data. (c) Heatmap of dataset-level CMS across integration methods (columns) and datasets (rows), stratified by simulator (panels). (d) Heatmap of Spearman's rank correlation ($\rho$) between CMS across datasets and integration methods.

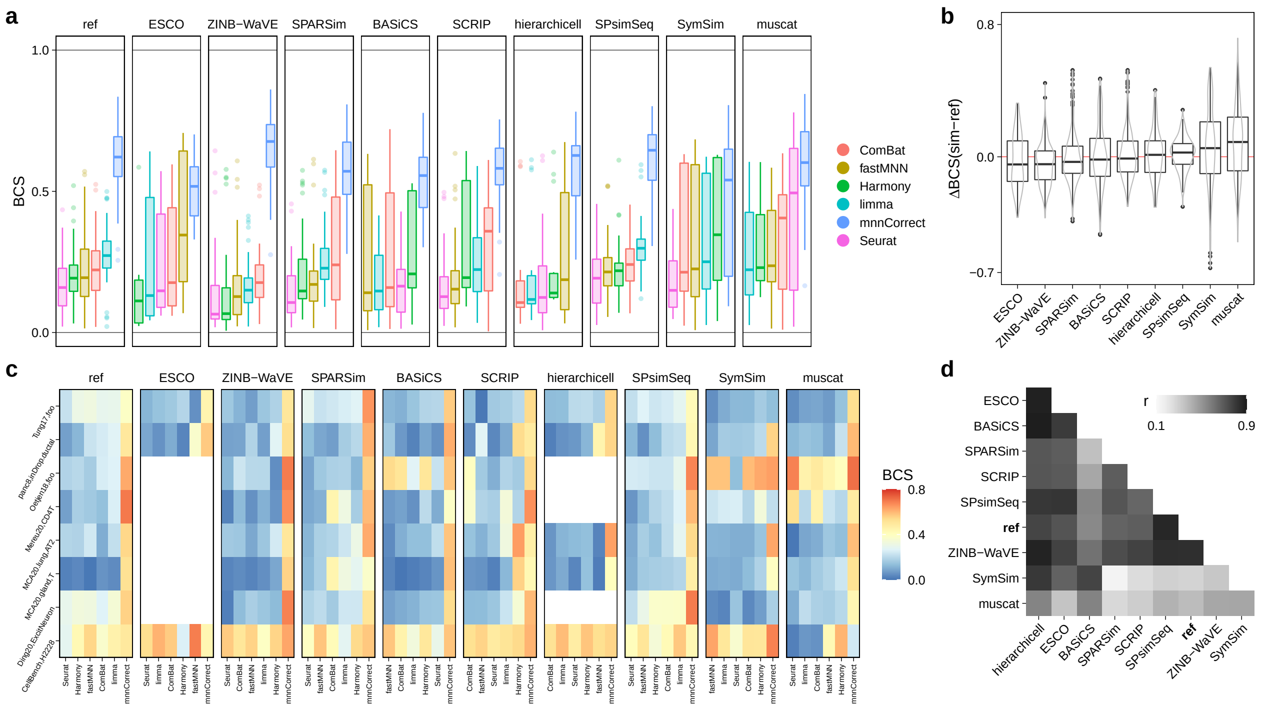


**Fig. S16:** Comparison of integration results across (experimental) reference and (synthetic) simulated data. (a) Boxplot of batch-level batch correction scores (BCS) across all type $b$ references, simulation and integration methods. (b) Boxplot of difference ($\Delta$) in batch-level BCS obtained from *ref*erence and *sim*ulated data. (c) Heatmap of dataset-level BCS across integration methods (columns) and datasets (rows), stratified by simulator (panels). (d) Heatmap of Spearman's rank correlation ($\rho$) between BCS across datasets and integration methods.

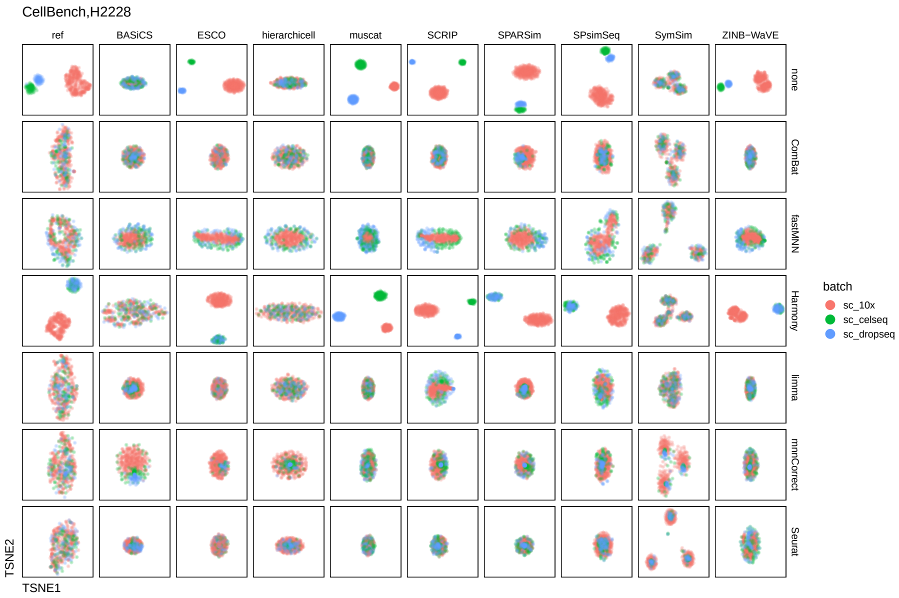


**Fig. S17:** Comparison of dimension reduction plots across simulators and integration methods for the *CellBench,H2228* dataset; points (cells) are colored by batch.

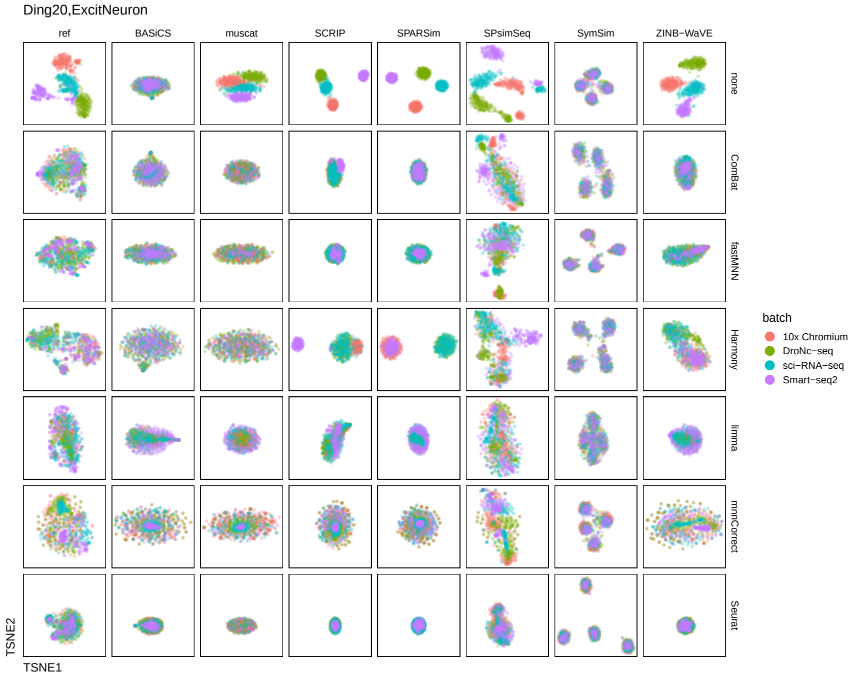


**Fig. S18:** Comparison of dimension reduction plots across simulators and integration methods for the *Ding20,ExcitNeuron* dataset; points (cells) are colored by batch.

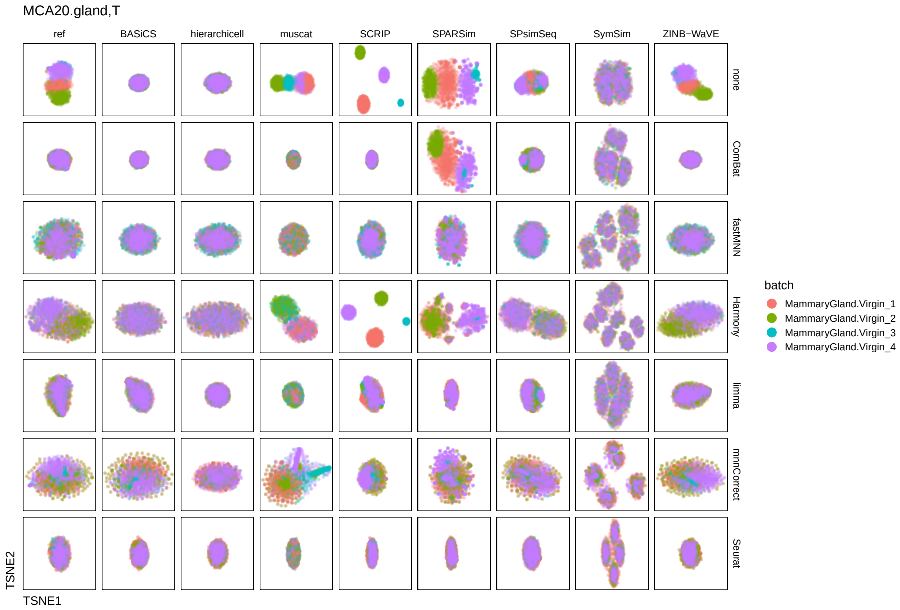


**Fig. S19:** Comparison of dimension reduction plots across simulators and integration methods for the *MCA20.gland,T* dataset; points (cells) are colored by batch.

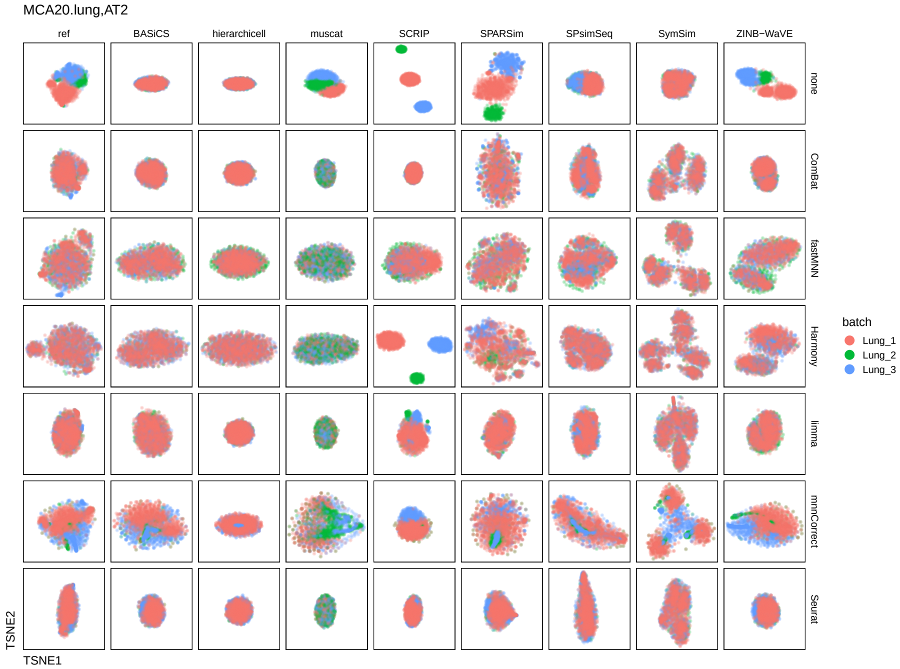


**Fig. S20:** Comparison of dimension reduction plots across simulators and integration methods for the *MCA20,lung,AT2* dataset; points (cells) are colored by batch.

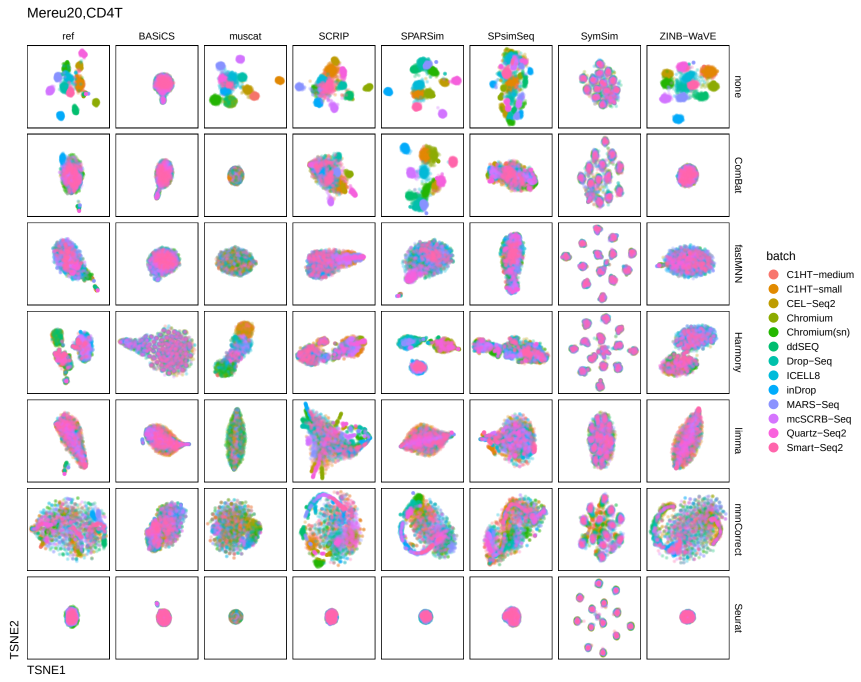


**Fig. S21:** Comparison of dimension reduction plots across simulators and integration methods for the *Mereu20,CD4T* dataset; points (cells) are colored by batch.

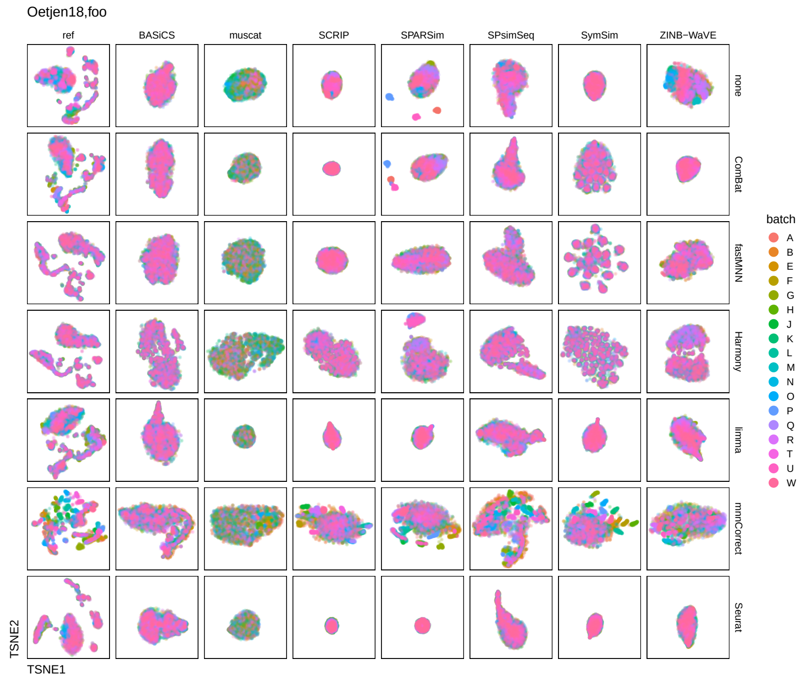


**Fig. S22:** Comparison of dimension reduction plots across simulators and integration methods for the *Oetjen18* dataset; points (cells) are colored by batch.

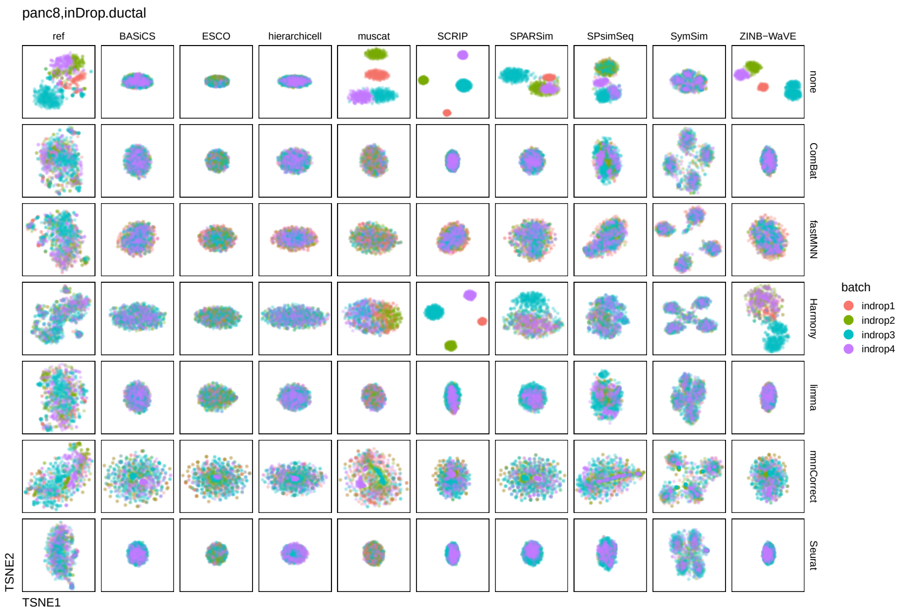


**Fig. S23:** Comparison of dimension reduction plots across simulators and integration methods for the *panc8,inDrop.ductal* dataset; points (cells) are colored by batch.

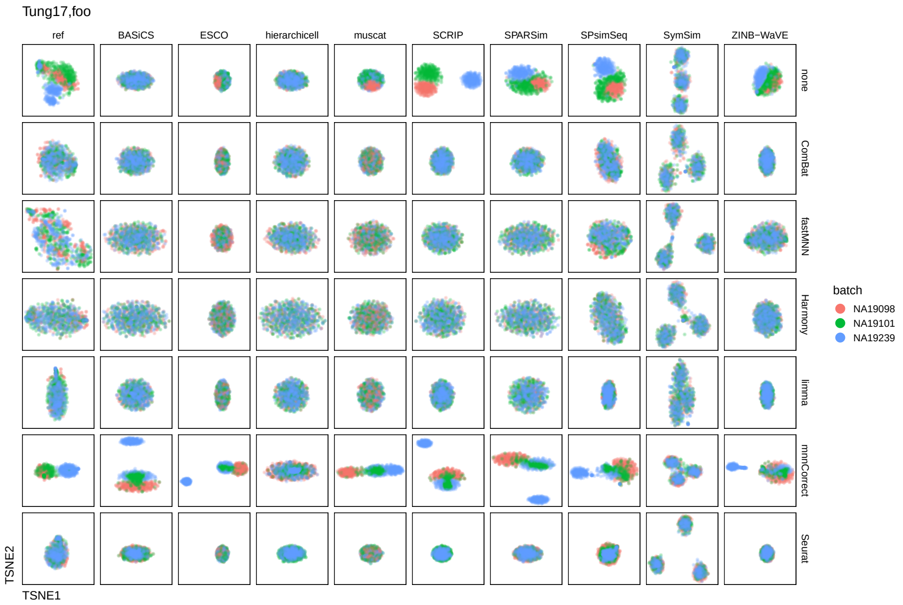


**Fig. S24:** Comparison of dimension reduction plots across simulators and integration methods for the *Tung17* dataset; points (cells) are colored by batch.

## 4.2 Summaries

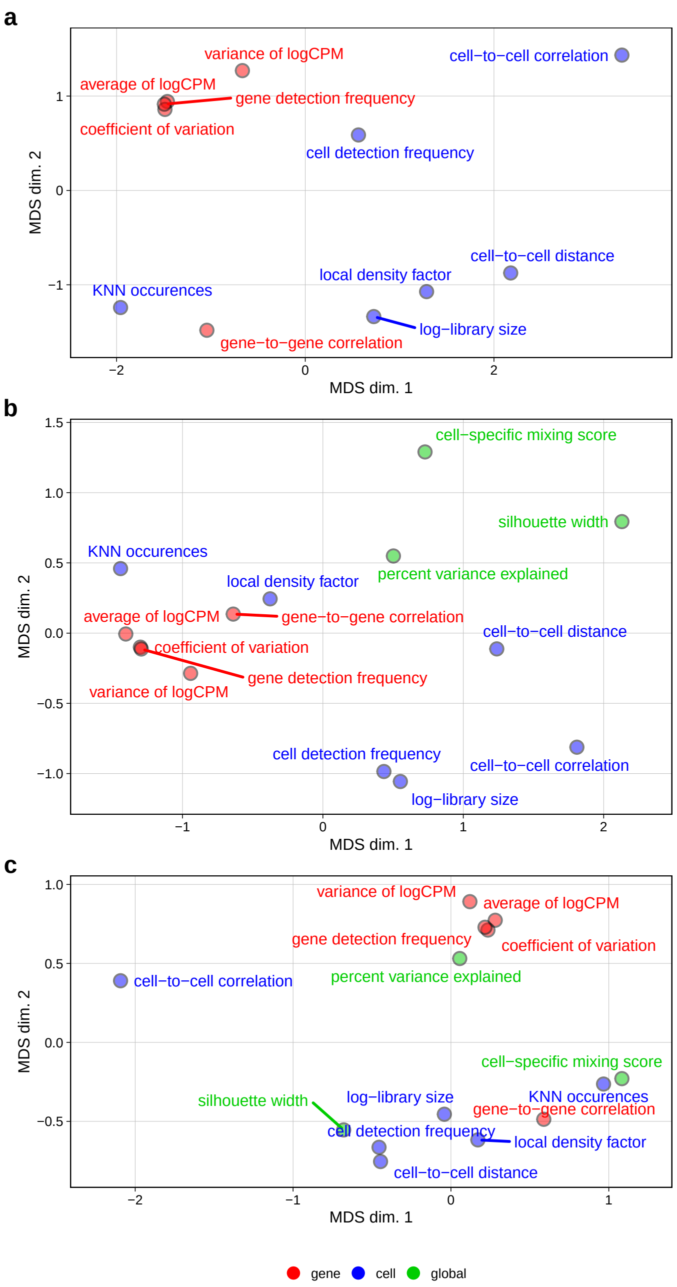


**Fig. S25:** Multi-dimensional scaling (MDS) plot of per-summary KS statistics across methods and datasets of type $n$ (a), $b$ (b), and $k$ (c). Summaries (points) are colored by their type: gene- (red), cell-level (blue) and, except for type $n$, global (green).

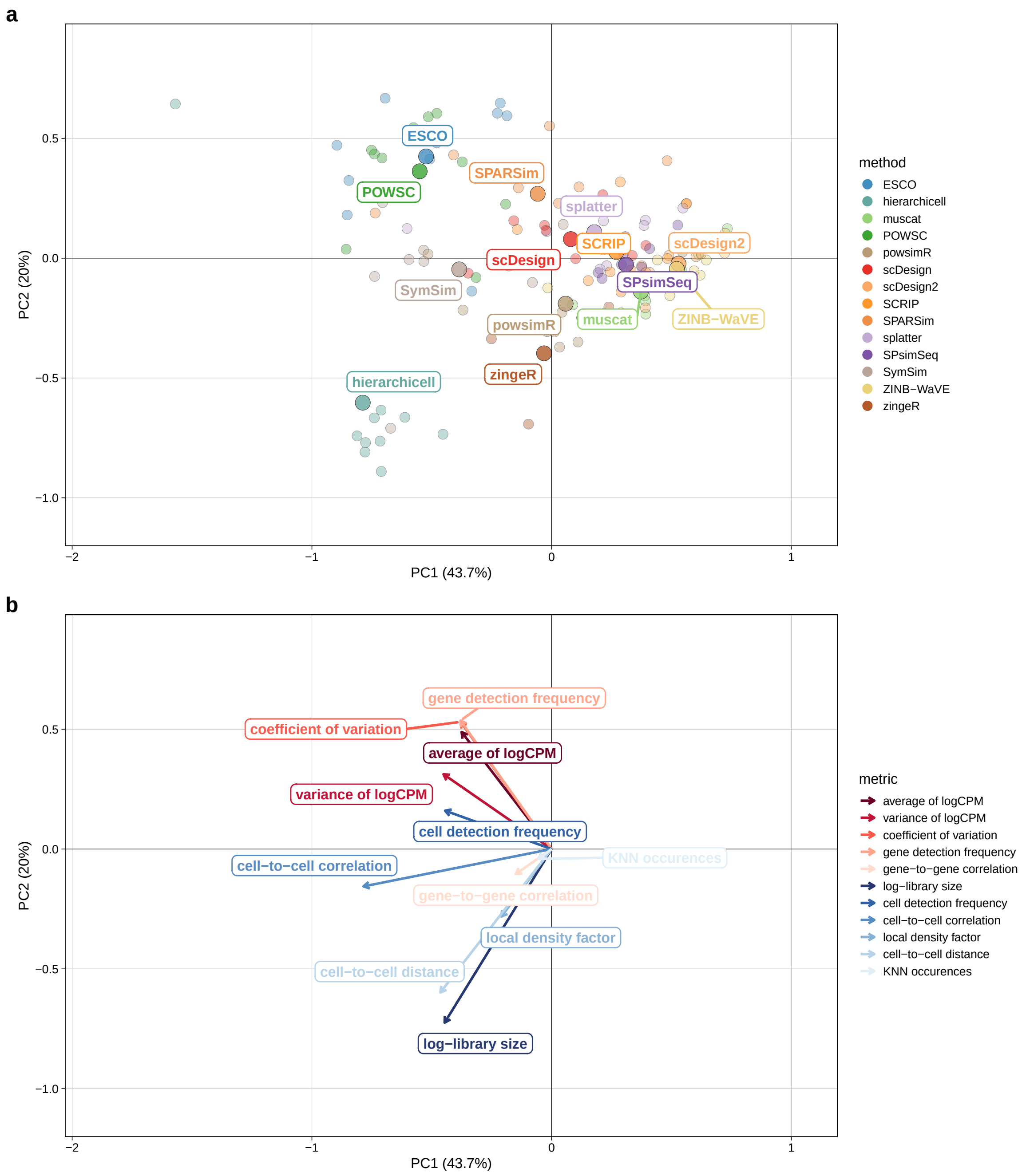


**Fig. S26:** Principal component (PC) analysis of KS statistics across summaries, methods, and datasets; type $n$. (a) First two PCs. Each small point corresponds to a dataset-method, large points represent per-method averages across datasets. Axis titles indicate the percentage of variance explained by each component. (b) PC loadings. Arrows correspond to summaries and are colored by type (gene- = red, cell-level = blue).

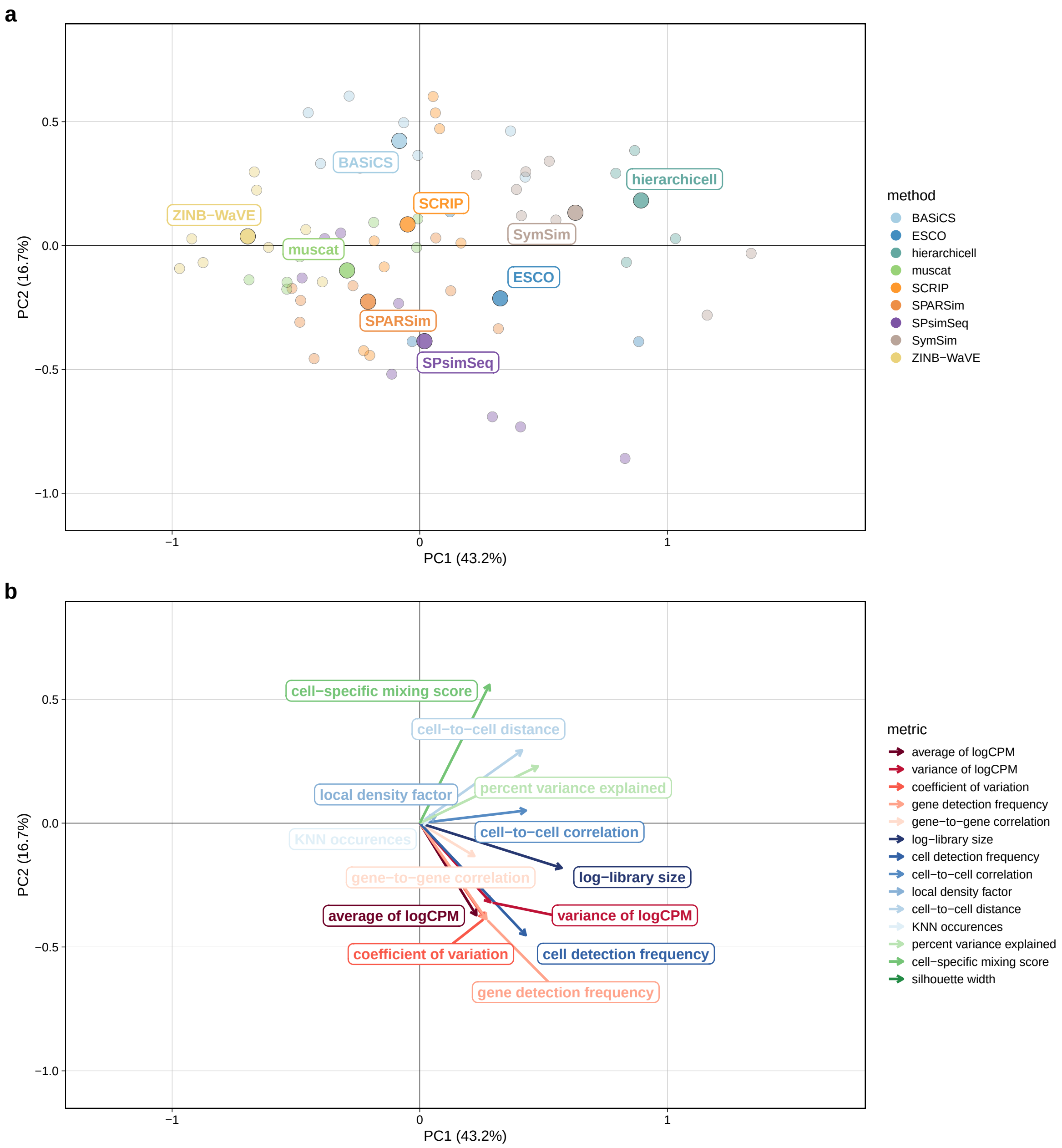


**Fig. S27:** Principal component (PC) analysis of KS statistics across summaries, methods, and datasets; type *b*. (a) First two PCs. Each small point corresponds to a dataset-method, large points represent per-method averages across datasets. Axis titles indicate the percentage of variance explained by each component. (b) PC loadings. Arrows correspond to summaries and are colored by type (global, gene- or cell-level).

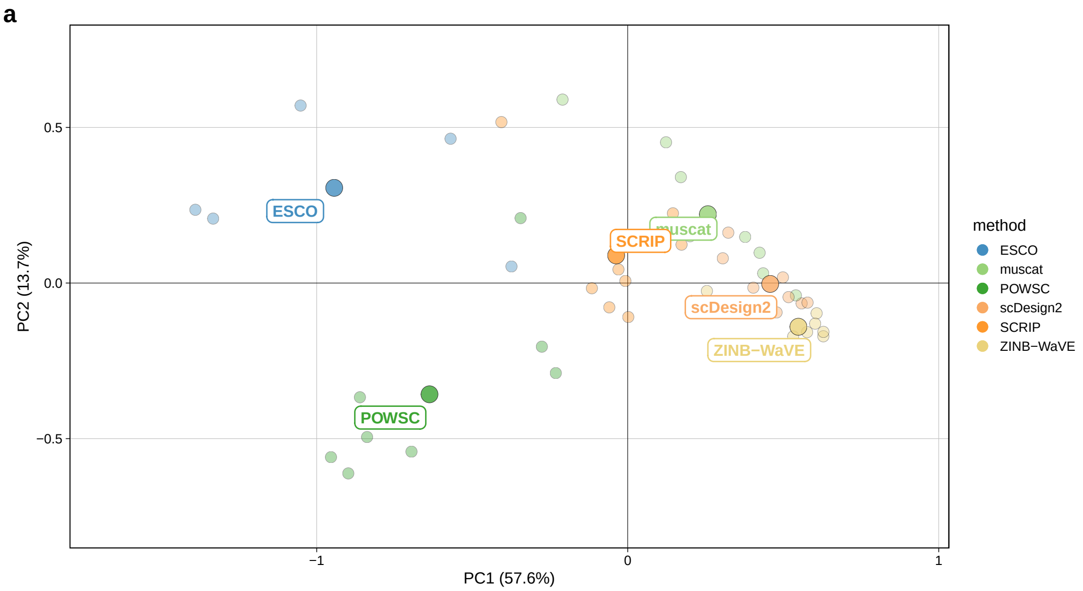


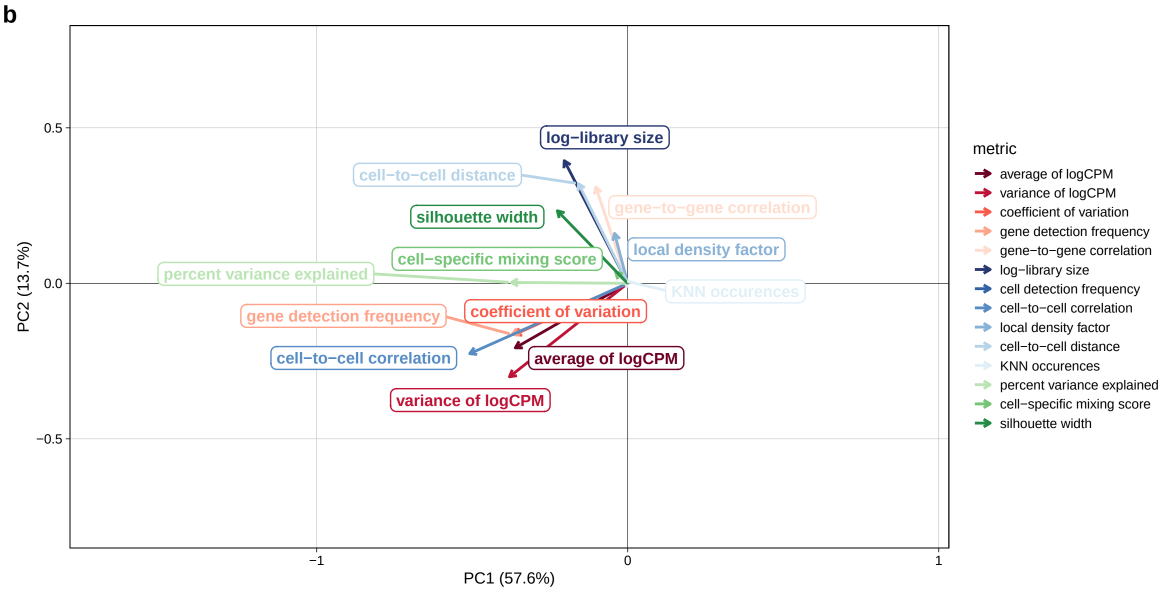


**Fig. S28:** Principal component (PC) analysis of KS statistics across summaries, methods, and datasets; type $k$. (a) First two PCs. Each small point corresponds to a dataset-method, large points represent per-method averages across datasets. Axis titles indicate the percentage of variance explained by each component. (b) PC loadings. Arrows correspond to summaries and are colored by type (gene- = red, cell-level = blue, global = green).
