# Supplementary material for: The shaky foundations of simulating single-cell RNA sequencing data
Source: Genome Biol. 2023 Mar 29;24:62. doi: 10.1186/s13059-023-02904-1 (PMC10061781; doi:10.1186/s13059-023-02904-1)
Supplement: Supplementary file 3 — Additional file 3. Review History. [file 13059_2023_2904_MOESM3_ESM.docx]

**1^st^ round**

**Reviewer 1**

In this work, the authors conducted a comprehensive benchmark of scRNA-seq simulators. This study is of critical importance to the prosperous single-cell field where many computational methods have been developed and require fair benchmarking by well-designed simulators.

Major comments:

1. An important criterion is not included in the comparison of simulators: whether or not a simulator can generate data with varying cell numbers and sequencing depths.

2. In section "KS test statistic between reference and simulated data across methods and summaries":
2.1. It seems unfair to combine cell-level comparison and cluster-/batch- level comparison results. While the global (cell-level) comparison calculates summary statistics from one simulation, batch- and cluster-level comparison uses repeated simulation for different numbers of times. I would recommend using the max{1+K, 1+B} for a coherent repetition number across different categories of comparisons.
2.2. In addition, I doubt the reliability of the K-S and Wasserstein statistics. The statistics are calculated from the repeated pairs between reference and simulated data for each summary. Hence, there is only one repetition for global comparison, thus one sample to calculate the K-S statistic. It's not reasonable to make any decision based on a one-sample-based statistic.

3. In Table 1, the model summary for SCRIP is incorrect.

4. Line 77. The summary of simulators in this paragraph is not very meaningful. Just because certain simulators are not used for a specific benchmark study doesn't mean that they cannot be used in the corresponding data analysis task. It could be just due to the choice of the author of these benchmark studies, or simply that some simulators have not been published yet.

5. What is the rationale behind transforming the gene expression from count to the log-CPM scale? It seems that many of the benchmark datasets used here are generated from the droplet-based protocols, and people have argued that for these types of datasets, just using the count values directly may be enough [1][2]. In particular, library size normalization may introduce unwanted artifacts [2]. Moreover, it seems that more than half of the considered simulators use count-value-based models like NB or ZINB. Will using the log-CPM scale still be suitable in this case? Comparison results should also be reported on the count scale.

6. In Figure 2, why do the boxplots in the two panels not seem to match each other?

7. I would suggest they add some metrics based on dimensionality reduction/2D visualization. I think this is the most intuitive way to show how similar the simulated data is to the real data.

Minor comments:

1. In Supp. Table 3, the descriptions for gene and cell detection frequency seem to be reversed.

2. Line 96. Are there three summary statistics here, instead of two? Since there are PVE, the silhouette width, and CMS.

3. Line 110. "deviance in cell-to-cell correlation" Since deviance is a goodness-of-fit statistic, it could be changed to "deviation".


Reference
[1] Svensson, V. Droplet scRNA-seq is not zero-inflated. Nat Biotechnol 38, 147-150 (2020). https://doi.org/10.1038/s41587-019-0379-5
[2] Townes, F.W., Hicks, S.C., Aryee, M.J. et al. Feature selection and dimension reduction for single-cell RNA-Seq based on a multinomial model. Genome Biol 20, 295 (2019). <https://doi.org/10.1186/s13059-019-1861-6>

**Reviewer 2**

This paper provides a comparison of methods for simulations single-cell RNA sequencing data. The authors give a good introduction to the strengths and weaknesses of simulations for developing and benchmarking computational analysis methods. The comparison to reference datasets is comprehensive and includes 16 methods, 12 reference datasets, scenarios with multiple batches and clusters, global, gene and cell level metrics and relationships between individual scores. Simulations are also evaluated as to whether they produce similar benchmarking results as the reference datasets for integration and clustering tasks. I have some suggestions which I hope will improve the paper but overall I find it to be an extremely well written and thorough exploration of the topic which will be valuable to researchers in the field.

Major concerns

My only major concern is around how the description of how the simulation methods were run. The methods sections for this were very brief, basically stating that default parameters were used. It is possible to get some more detail from the code but this requires understanding a fairly complex pipeline. More specifically I would like to see information about how required user-supplied parameters were set. As shown in your table user input is required to generate some of the scenarios tested for some methods but it is not clear which parameters were set in those cases or how values were chosen.

Minor concerns

* It might be good to mention in the introduction that you have chosen to exclude continuous scenarios and methods that produce those. This is well described in the discussion but I spent the whole paper until then wondering why they hadn't been mentioned.
* SCRIP has recently been published so would be good to include the reference for that https://doi.org/10.1093/bioinformatics/btab824. I think some of your other bioRxiv references may also be published now so maybe worth checking those (can be done just before this is published).
* It would be good to mention somewhere that Splatter contains multiple models and that the splat model is what you have evaluated here.
* I was sometimes a bit unclear about what you considered a "reference-based simulation", is it when parameters are estimated from a real dataset, when the simulation can only generate the structure in the reference, when genes can be linked to the reference? It would also helpful to label the compared methods as to which category you think they belong to.
* In a few places you mention that Splatter simulates batches separately and concatenates the results, is this correct? My understanding was that batch effect factors were introduced into the model at an early stage and carried through the simulation to generating counts. This is an example of something that could maybe be answered by having more detail on how the simulations were run.
* Figure 3 would be clearer if there were labels showing what each of the panels are and what the red/blue/green areas show rather than having to refer the the caption.
* Did you also record memory usage in your scalability testing? This would be interesting to see as it could often be the limiting factor rather than time for big simulations.
* In the discussion I was unsure whether you consider flexibility introduced by user parameters to be a strength or weakness of a model? At one stage you state that "most current simulators are rather limited in their applicability" because they require users to define groups/batches but in other parts of the paper you point out that simulations that stick too closely to the reference have limited uses.
* Similarly I wasn't quite sure whether you think it would be desirable to have an optimal "all-rounder" simulation or if you think it would be preferable/more useful for simulations to be tailored to the task they are being used to assess.
* You say that current methods are limited in complexity of the simulations that can generate but there are several methods which can simulate combinations of groups and batches which would seem to cover most cases. Am I misunderstanding or is there another type of complexity you mean here?
* I really like your comment that comparisons between simulations and references should be included in benchmarks. I would suggest that this could be expanded to any time a simulation is used or proposed :-).
* For the metrics it wasn't clear for some of them (like coefficient of variation) if they were calculated on logCPM or raw counts (or some other transformation)
* For the more complex metrics (LDF, cell-cell distance, KNN, PVE, CMS) it would be good to have a brief description in the methods (and maybe where they are first mentioned) as these are unlikely to be familiar to the reader. Doesn't need the technical detail, just a suggestion of what they are measuring.
* In general I felt that some of the (excellent) detail in the supplementary methods could be included in the main methods section where the reader is more likely to see it.
* In supplementary figure 11 (runtimes) I would maybe try an alternative to bar charts as the log scale makes it look as though some methods have negative runtimes. Maybe a line chart would work? If you wanted to further quantify this you could fit a linear model to each method and use the slope as a score for scalability.
* You mention the Cao et al. SimBench paper as the only previous independent evaluation of scRNA-seq simulators. It would be interesting to include in the discussion where your results agree/disagree with theirs given you have compared many of the same methods.
* Did you consider including any of the metrics from CountSimQC/SimBench given that these packages already exist for this kind of comparison? If there are reasons you prefer the metrics shown here it would be good to include that somewhere.
* It would be good to include the version of the packages used in the paper. This is important for future reader who may want to check if there have been updates since this evaluation.
* Do you have a table of all the other methods you found but chose to exclude from the evaluation? If so it would be nice to include that as a supplementary to show what is out there.

Software/code

* Thank you for providing the all the code used for your evaluation! This will be invaluable for anyone else wanting to do something similar and is an excellent example what should be provided for reproducibility.
* One thing I couldn't find were the JSON configuration files in the meta/ directory. These would be needed by anyone wanting to run the pipeline. If there is a reason you can't provide the exact files you used please at least provide an example so people can see the format.

As I said above I feel that this is already a very high quality paper. Many of my comments are suggestions which you may which you may choose not to change or questions I personally found interesting from what you have shown.

**Authors’ response**

Reviewer #1

In this work, the authors conducted a comprehensive benchmark of scRNA-seq simulators. This study is of critical importance to the prosperous single-cell field where many computational methods have been developed and require fair benchmarking by well-designed simulators.
Major:
1. An important criterion is not included in the comparison of simulators:
whether or not a simulator can generate data with varying cell numbers and sequencing depths. Regarding ”varying cell numbers”: The number of cells may be, in principle, varied for any simulation by either downsampling (less cells) or repeated simulation (more cells). Most simulators also offer arguments for this, but note that this would not affect the computed summaries.
Regarding “varying [...] sequencing depth”: Here, we set out to compare reference-based simulations. By definition, the reference data’s depth should thus be retained in order to make comparisons between reference and simulation and thus validate a method’s faithfulness. There are indeed many other aspects that could be of interest that we did not include (e.g., varying the number of batches/clusters; altering inter-batch/-cluster differences, i.e., the fraction of DE genes and magnitude of DE, etc.). However, usage of any such parameters would make it challenging to validate the faithfulness of the generated data because simulations would be (in part or fully) de novo. We would be happy to discuss the possibility of including more aspects in a qualitative way (e.g., as additional columns in Table 1). However, we would argue that flexibility does not indicate usefulness, and marking methods as highly flexible without validation would be misleading (please see also our response to Reviewer 2’s comment on flexibility vs. applicability).

2. In section "KS test statistic between reference and simulated data across methods and summaries":
2.1. It seems unfair to combine cell-level comparison and cluster-/batch-level comparison results. While the global (cell-level) comparison calculates summary statistics from one simulation, batch- and cluster-level comparison uses repeated simulation for different numbers of times. I would recommend using the max{1+K, 1+B} for a coherent repetition number across different categories of comparisons.
We are not sure we fully understand. Perhaps to clarify: Global summaries are computed once per reference-simulation. Gene- and cell-level summaries are computed once per batch (for type b) or cluster (for type k). Thus, as there is a fixed number of batches/clusters for a given reference, there is a fixed number of summaries per reference-simulation pair. Simulations themselves are not “repeated”; there is precisely one simulation per method and reference. Thus, for all simulators of a given type, the same number of statistics is obtained.

2.2. In addition, I doubt the reliability of the K-S and Wasserstein statistics. The statistics are calculated from the repeated pairs between reference and simulated data for each summary. Hence, there is only one repetition for global comparison, thus one sample to calculate the K-S statistic. It's not reasonable to make any decision based on a one-sample-based statistic.
Indeed, we are summarizing the similarity of the distribution of each reference-simulation pair for each summary (e.g., coefficient of variation, local density factor) in a single number, either the K-S or Wasserstein statistic. But, this is repeated for 12 reference datasets and a variety of subsets of the datasets. So, no decisions are based on a “one-sample-based” statistic.

3. In Table 1, the model summary for SCRIP is incorrect.

Indeed, the model description was mistakenly copied from scDesign2.

This has been changed to ‘(Beta-)Gamma-Poisson’.

4. Line 77. The summary of simulators in this paragraph is not very meaningful. Just because certain simulators are not used for a specific benchmark study doesn't mean that they cannot be used in the corresponding data analysis task. It could be just due to the choice of the author of these benchmark studies, or simply that some simulators have not been published yet.

We agree that the frequency with which simulators are applied in benchmarking specific analysis tasks does not indicate they cannot be used in general. However, the purpose of this paragraph and the corresponding ‘Benchmark(s)’ column in Table 1 is to highlight how widely (in terms of tasks) and frequently each simulator has been applied in current publications, which we believe to be an informative observation.

5. What is the rationale behind transforming the gene expression from count to the log-CPM scale? It seems that many of the benchmark datasets used here are generated from the droplet-based protocols, and people have argued that for these types of datasets, just using the count values directly may be enough [1][2]. In particular, library size normalization may introduce unwanted artifacts [2]. Moreover, it seems that more than half of the considered simulators use count-value-based models like NB or ZINB. Will using the log-CPM scale still be suitable in this case? Comparison results should also be reported on the count scale.
It is true that any form of transformation makes certain assumptions and might introduce unwanted effects. However, various summaries that we use are simply not applicable to raw counts (e.g., variance or PCA) and therefore require “expression” values. In addition, because of heteroskedasticity, it is also not appropriate to calculate some summaries (e.g., average, variance) on the linear scale.

6. In Figure 2, why do the boxplots in the two panels not seem to match each other?
We are unsure why they would appear to not match. Both panels were generated from the same data (statistics across all references, simulations and summaries), but stratified differently (by summary (a) and method (b), respectively). To give two examples: Global summaries (e.g., silhouette width) are missing for simulators that can only accommodate type n in (a) as well as (b); ZINB-WaVE and scDesign2 are amongst the left-most methods across various summaries in (a) and give low statistics in (b); etc.

7. I would suggest they add some metrics based on dimensionality reduction/2D visualization. I think this is the most intuitive way to show how similar the simulated data is to the real data.
We would like to note that cell-mixing scores (CMS), difference in local density factor (ΔLDF), number of KNN occurrences and cell-to-cell distance are computed in PCA space, which is a linear dimensionality reduction. Meanwhile, we would assume that any metric computed on a non-linear space (e.g., t-SNE and UMAP representation) would not yield a more faithful data summary, as our current set of summaries (that are not based on PCA) leverages the full set of features rather than a lower-dimensional representation.

Minor:
1. In Supp. Table 3, the descriptions for gene and cell detection frequency seem to be reversed.
They are in fact correct, but we understand how this might be confusing at first glance. Gene detection frequency is the fraction of detected cells (for a given gene), while cell detection frequency is the fraction of detected genes (for a given cell). We have clarified these descriptions as follows: gene detection frequency: fraction of cells with non-zero count (for a given gene) cell detection frequency: fraction of detected genes (for a given cells)

2. Line 96. Are there three summary statistics here, instead of two? Since there are PVE, the silhouette width, and CMS.
Indeed, thank you for noticing this; fixed.

3. Line 110. "deviance in cell-to-cell correlation" Since deviance is a goodness-of-fit statistic, it could be changed to "deviation".
Done.

Reference:
[1] Svensson, V. Droplet scRNA-seq is not zero-inflated. Nat Biotechnol 38, 147-150 (2020).
https://doi.org/10.1038/s41587-019-0379-5
[2] Townes, F.W., Hicks, S.C., Aryee, M.J. et al. Feature selection and dimension reduction for single-cell RNA-Seq based on a multinomial model. Genome Biol 20, 295 (2019). https://doi.org/10.1186/s13059-019-1861-6

Reviewer #2
This paper provides a comparison of methods for simulations single-cell RNA sequencing data. The authors give a good introduction to the strengths and weaknesses of simulations for developing and benchmarking computational analysis methods. The comparison to reference datasets is comprehensive and includes 16 methods, 12 reference datasets, scenarios with multiple batches and clusters, global, gene and cell level metrics and relationships between individual scores. Simulations are also evaluated as to whether they produce similar benchmarking results as the reference datasets for integration and clustering tasks. I have some suggestions which I hope will improve the paper but overall I find it to be an extremely well written and thorough exploration of the topic which will be valuable to researchers in the field.
Major:
My only major concern is around how the description of how the simulation methods were run. The methods sections for this were very brief, basically stating that default parameters were used. It is possible to get some more detail from the code but this requires understanding a fairly complex pipeline. More specifically I would like to see information about how required user-supplied parameters were set. As shown in your table user input is required to generate some of the scenarios tested for some methods but it is not clear which parameters were set in those cases or how values were chosen.
Regarding “user input is required to generate some of the scenarios tested”: While we state that simulation types marked with (✓) required parameter inputs, we do not test these as they are de novo (not reference-based) simulations (e.g., scDesign is marked as ✓/(✓) for type b/k, but included here as a type n and b method only). While it is in fact true, we understand how the statement “default parameters were used” might be unsatisfactory. However, it is not straightforward – and, in our opinion, not useful – to list (and possibly explain) each method’s function call (for both, estimation and simulation). For each method, this would result in a ~0.5 page paragraph scattered with (often not very meaningful) parameter names (we had originally written this out). Thus, we strongly believe that the best source for how a method was run is the actual code. Specifically, the Snakemake workflow organization and R script naming schemes are described in detail in the GitHub repository’s README (note also that a snapshot of the GitHub Repository is posted on Zenodo; the DOI is listed in the manuscript). This should make it easy for readers to see how methods were run exactly (e.g., parameter estimation wrappers are under 03-est_pars-<method>.R, simulation wrappers are under 04-sim_data-<method>.R etc.). To highlight this, we have added in the Method’s “Code availability” section that: Workflow structure, code organization and script naming schemes are described in more detail on the repository's landing page.”

Minor:
* It might be good to mention in the introduction that you have chosen to exclude continuous scenarios and methods that produce those. This is well described in the discussion but I spent the whole paper until then wondering why they hadn't been mentioned.
Good point. We have added the following statement in the results’ “Benchmark design” section: Notably, there are various methods aimed at simulating continuous scenarios (e.g., a trajectory or time-course). Here, we limited comparisons to methods that generate a single group or multiple groups of cells as current continuous simulators are fully or in part de novo, making it challenging to validate the faithfulness of the data they generate (see Discussion).

* SCRIP has recently been published so it would be good to include the reference for that https://doi.org/10.1093/bioinformatics/btab824. I think some of your other bioRxiv references may also be published now so maybe worth checking those (can be done just before this is published).
Thank you for mentioning this. We checked all bioRxiv references and updated them where applicable (ESCO, SCRIP and scDesign2); the only yet unpublished method is zingeR.

* It would be good to mention somewhere that Splatter contains multiple models and that the splat model is what you have evaluated here.
Good point. We have added a note to Splatter in Table 1 mentioning this.

* I was sometimes a bit unclear about what you considered a "reference-based simulation", is it when parameters are estimated from a real dataset, when the simulation can only generate the structure in the reference, when genes can be linked to the reference? It would also be helpful to label the compared methods as to which category you think they belong to.
In the Introduction, we state that “reference-based methods estimate parameters to mimic the gene expression profiles observed in the reference dataset”, i.e., we consider a reference-based simulation one where i) parameters are estimated from a real dataset; and, ii) respective data is mimicked. Whether or not genes can be linked between reference and simulation is method dependent, but irrelevant for the distinction between reference-based and de novo simulation.

* In a few places you mention that Splatter simulates batches separately and concatenates the results, is this correct? My understanding was that batch effect factors were introduced into the model at an early stage and carried through the simulation to generating counts. This is an example of something that could maybe be answered by having more detail on how the simulations were run.
We apologize for the confusion here. It is true that Splatter is capable of incorporating batch-/cluster-specific factors in the model, however, only for de novo simulations. Specifically, there are various parameters for this that may be specified by the user, but cannot be estimated from the data. Hence, we labeled Splatter as type n: ✓ but type b/k: (✓) (see also Table 1’s caption), and resorted to concatenating independent type n simulations for type b/k data.

* Figure 3 would be clearer if there were labels showing what each of the panels are and what the red/blue/green areas show rather than having to refer to the caption.
Done.

* Did you also record memory usage in your scalability testing? This would be interesting to see as it could often be the limiting factor rather than time for big simulations.
We agree that this is useful information and have now recorded memory usage (Residual Set Size in MBs); see two new Supp. Figs. (after the one depicting runtimes): i) visualizing memory usage analogous to runtimes (barplot); and, ii) plotting runtimes vs. memory usage across gene/cell subsets (line plot).

* In the discussion I was unsure whether you consider flexibility introduced by user parameters to be a strength or weakness of a model? At one stage you state that "most current simulators are rather limited in their applicability" because they require users to define groups/batches but in other parts of the paper you point out that simulations that stick too closely to the reference have limited uses.
We share your uncertainty regarding this point: arguably, it cannot be clearly determined whether flexibility is a strength or weakness; otherwise put: high flexibility does not necessarily mean it is useful. For example, various methods provide a large number of parameters to alter different aspects of the simulation. However, since these are reliant on user-inputs, it is challenging to evaluate how well they recapitulate real data characteristics. Our discussion tries to convey the point that current reference-based simulators are “rather limited” (but we can demonstrate their non-/faithfulness), while de novo simulators offer great yet arbitrary flexibility (that cannot be validated). In brief, we believe limited applicability to be more useful than wide but unreliable applicability; ideally, of course, one would have both (see next point).

* Similarly I wasn't quite sure whether you think it would be desirable to have an optimal "all-rounder" simulation or if you think it would be preferable/more useful for simulations to be tailored to the task they are being used to assess.
Of course an “all-rounder” would be most desirable, i.e., a method that is capable of accommodating any design (e.g., to give an extreme example, multiple clusters with cluster-specific batch effects across multiple experimental conditions). Currently, however, most simulators are not capable of faithfully producing an arbitrary design, and are focused on a specific task such as clusters, batch effects etc.

* You say that current methods are limited in the complexity of the simulations that they can generate but there are several methods which can simulate combinations of groups and batches which would seem to cover most cases. Am I misunderstanding or is there another type of complexity you mean here?
We understand how the points made might sound contradicting. Perhaps to clarify: i) 2/16 methods are pure type n;

ii) there are 10/16 and 8/16 type b and k methods, respectively; and, first and foremost, iii) the fact that a given method supports both b and k does not mean it can do both at the same time. Specifically, to our knowledge, only muscat can estimate parameters from a multi-batch multi-cluster dataset (i.e., accommodate batch effects across multiple clusters). Thus, overall, we believe the statement “limited in the complexity” to be accurate.

* I really like your comment that comparisons between simulations and references should be included in benchmarks. I would suggest that this could be expanded to any time a simulation is used or proposed :-).
Thank you for the suggestion. We have expanded our Discussion statement as follows:
Having such reference-simulation comparisons available every time that a simulator is proposed or used, as well as in every (non-neutral) benchmark, would add credibility to the results.

* For the metrics it wasn't clear for some of them (like coefficient of variation) if they were calculated on logCPM or raw counts (or some other transformation)
We define X / Y as counts / expressions (= logCPM) in the introductory paragraph of Supp. Text section ‘2 Quality control summaries’ proceeding Supp. Table 3. Here, any summary that mentions ‘expression’ uses logCPM (e.g., CV = √σ/μ where σ = variance and μ = mean are of expression); the PVE states ‘fraction of expression variance’; cell-to-cell distance, KNN occurrences and silhouette width use PCA space, which is in turn computed on Y.
Finally, the CMS and ΔLDF use PCA space as well. We have clarified this by adding “...neighborhood (in PCA space)” in their description as this was previously not included in the table.

* For the more complex metrics (LDF, cell-cell distance, KNN, PVE, CMS) it would be good to have a brief description in the methods (and maybe where they are first mentioned) as these are unlikely to be familiar to the reader.
Doesn't need the technical detail, just a suggestion of what they are measuring.
We have expanded on the description of quality control summaries in the Methods section, and added appropriate references for the CMS, LDF and PVE.

* In general I felt that some of the (excellent) detail in the supplementary methods could be included in the main methods section where the reader is more likely to see it.
While we had considered this, we believe it is challenging to do. Specifically, it is a subjective endeavor to decide which information is most relevant. For example, expanding on only a subset of summaries would be based on what we feel is intuitive; explaining certain parts of the workflow in detail might be misleading etc. Thus, we felt that pointing to the corresponding section in the supp. text to be the most neutral and clear approach. Nevertheless, we understand the point made by the reviewer and would be happy to discuss this further (e.g., which details in specific could/should be included).

* In supplementary figure 11 (runtimes) I would maybe try an alternative to bar charts as the log scale makes it look as though some methods have negative runtimes. Maybe a line chart would work? If you wanted to further quantify this you could fit a linear model to each method and use the slope as a score for scalability.
Agreed, the y-axis was indeed not ideal. To make the runtime barplots clearer, we have i) changed from log- to sqrt-scale on the y-axis; and, ii) reordered methods according to their average overall runtimes across all gene and cell subsets (such that slow/fast methods are more easily identified). Regarding a possible scoring of runtimes / memory usage: First of all, we think that fitting any model to the data presented here would be excessive. There are 5 replicates per gene-/cell-subset, 4 subsets each, and thus, 20 data points across genes and cells, respectively.

* You mention the Cao et al. SimBench paper as the only previous independent evaluation of scRNA-seq simulators. It would be interesting to include in the discussion where your results agree/disagree with theirs given you have compared many of the same methods.
We have expanded briefly in the Discussion on how our benchmark design and results compare to those presented in Cao et al. (see highlighted text in the MS).

* Did you consider including any of the metrics from CountSimQC/SimBench given that these packages already exist for this kind of comparison? If there are reasons you prefer the metrics shown here it would be good to include that somewhere.
To the best of our knowledge, we have included the vast majority of the summaries provided by the countsimQC

and SimBench packages. The choice to implement all summaries in stand-alone R scripts was motivated as follows: i) summaries can be computed for different groups of cells (globally, per-batch/-cluster); and, ii) addition/removal and modification of summaries is more flexible and will only trigger re-computation of affected results (via Snakemake). Finally, our benchmark design allows for easily extending the most commonly used summaries (e.g., from countsimQC and SimBench) to include additional ones – by us (e.g., CMS, ΔLDF, KNN occurrences etc.) and anyone interested in building on or using (in part or full) our framework in the future..

* It would be good to include the version of the packages used in the paper. This is important for future readers who may want to check if there have been updates since this evaluation.
All package versions are listed in the session_info.txt file (available at DOI:10.5281/zenodo.5678872) that captures the output of sessionInfo() upon loading all packages used throughout the workflow (see code/10-session_info.R script).

* Do you have a table of all the other methods you found but chose to exclude from the evaluation? If so it would be nice to include that as a supplementary to show what is out there.
As mentioned in the first results section ”Benchmark design”, we based our method-selection on the scRNA-seq tools database at https://www.scrna-tools.org (>1k methods) where we i) filtered for methods holding a “simulation” tag (65 methods); and, ii) selected methods that were reference-based as well as installable and runnable. While we have compiled a table containing all simulators (including the ~40 not selected), we have chosen not to include it since i) the majority of methods were GitHub-based, not maintained and/or not installable/runnable; ii) about a dozen methods were for simulating trajectories only; and, iii) the remainder of methods was simply not reference-based, i.e., fully reliant on parameter inputs. Thus, all “useful” methods (runnable and reference-based) were in fact included in our comparison. And, in fact, a complete list of methods can be viewed directly in the scRNA-Seq tools database.
Software/code:

* Thank you for providing all the code used for your evaluation! This will be invaluable for anyone else wanting to do something similar and is an excellent example of what should be provided for reproducibility.
Thank you for noticing and the encouraging comment!

* One thing I couldn't find were the JSON configuration files in the meta/ directory. These would be needed by anyone wanting to run the pipeline. If there is a reason you can't provide the exact files you used please at least provide an example so people can see the format.
Correct and our apologies. These were mistakenly not pushed to GitHub, and currently would prevent reproducing the study as it is. We have now added them to the repository.

As I said above I feel that this is already a very high quality paper. Many of my comments are suggestions which you may choose not to change or questions I personally found interesting from what you have shown.

**2^nd^ round**

**Reviewer 1**

The authors responded to my comments but made no changes in the revised manuscript. I still think the following three comments need to be addressed with changes.

1. An important criterion is not included in the comparison of simulators:
whether or not a simulator can generate data with varying cell numbers and sequencing depths.

I would like to clarify that some simulators regard cells as random observations, while other simulators regard pairs of (cell, gene) as variables. For the latter simulators, such as ZINB-WaVE, the simulated count matrix would have the same dimensions as the input real count matrix; hence, how to do "repeated simulation (more cells)" (the authors' reply) is not as obvious as it seems. I would suggest that the authors add a column in Table 1 about what are regarded as variables and observations by each simulator.


4. Line 77. The summary of simulators in this paragraph is not very meaningful. Just because certain simulators are not used for a specific benchmark study doesn't mean that they cannot be used in the corresponding data analysis task. It could be just due to the choice of the author of these benchmark studies, or simply that some simulators have not been published yet.

I still don't think the benchmark column in Table 1 provides useful information. The summary of existing benchmark studies only provides limited information regarding the popularity of the simulators and would favor older simulators, which are not necesarily better. In other words, this column should be highly correlated with the publication years of simulators.

At least I would suggest the authors to change the text to reflect this possible bias.


5. What is the rationale behind transforming the gene expression from count to the log-CPM scale? It seems that many of the benchmark datasets used here are generated from the droplet-based protocols, and people have argued that for these types of datasets, just using the count values directly may be enough [1][2]. In particular, library size normalization may introduce unwanted artifacts [2]. Moreover, it seems that more than half of the considered simulators use count-value-based models like NB or ZINB. Will using the log-CPM scale still be suitable in this case? Comparison results should also be reported on the count scale.

The authors's response is about the inapplicability of several critera to raw counts. However, even though linear scale may be inappropriate, simply using the log(1 + count) transformation, without using library size normalization should be enough. This can reduce the issue of heteroskedasticity without introducing additional artifacts. Moreover, the authors should emphasize in the text that they used the same transformation for all simulators for a fair comparison. I also suggest the authors to add a discussion about the relative advantages and disadvantages of using raw counts, CPM, log(1 + count), and log-CPM.

**Reviewer 2**

Thank you for your responses to my comments, they were very clear and addressed my questions. I think I now have a much better understanding of how you performed the evaluation and your position of things such as the difference between "reference" and "de novo" simulations. I would suggest that these could be incorporated into the paper to clarify these points for future readers.

I still have some concerns about how the methods were used, particularly Splatter. From your comments I believe that you have simulated clusters and batches using Splatter by generating several independent datasets and concatenating them, rather than using the in built functionality for these scenarios. This may have affected the metrics you have calculated and your evaluations as two splat simulations are independent even if they use the same estimated parameters (i.e. Gene 1 in one simulation has no correspondence to Gene 1 in another simulation). I think your motivation for doing this is to cover these scenarios without setting a user-defined parameter however, you still use the number of clusters/batches in the real dataset to decide how many simulations to concatenate. Aside from the effect of this on the evaluation of Splatter I think it also disadvantages the other packages that can generate these scenarios with user input but for which you haven't employed this same workaround (powsimR and scDesign).

There are three suggested approaches I have for how to address this (there could be other approaches you decide to try):

1. Keep the current approach but extend it to these other packages and clearly state in the text how and why this has been done and that it is a non-standard way for these tools to be used (not my preference but maybe the least amount of work).
2. Use the inbuilt parameters for these scenarios for these packages. As I said you are already using these values from the real data so they could be passed directly to the packages rather than using this workaround.
3. Remove Splatter from the cluster and batch comparisons. This would be consistent with how the other packages are currently treated and your decision to focus on reference-based simulations.

It is possible that I have misunderstood your intentions or missed a technical reason why things had to be done this way. If that is the case please correct me.

Whatever your approach my main concern is that the packages are treated consistently and it is clear to the reader when you have done something outside normal usage. This concern extends to some of the current methods. While I understand your reluctance to exapnd the methods section it should be clear what changes you have made to method usage without having to search through the code. You state that "With few exceptions, methods were run using default parameters and, if available, following recommendations given by the authors in the corresponding software documentation" but there is no details of what these exceptions are. For example I noticed that in your script for powsimR you actually generate two populations and select one to use for evaluation but that is not explained to the reader who may not look at the code or have enough knowledge to interpret what it does or understand parameter names. I don't expect you to explain the details of every method but please describe when you have chosen to do something outside default usage. The supplementary methods you have for datasets and metrics are excellent but this detail is missing for the simulation methods.

**Authors’ response**

Amendments unrelated to reviewer comments:

- Under Results section “Batch simulators yield over-optimistic but faithful integration

method performance”, we wrongly referenced Supplementary Figure 14 depicting CMSs

instead of BCSs, which we did not include; this has been fixed.

Reviewer #1:

The authors responded to my comments but made no changes in the revised manuscript.

I still think the following three comments need to be addressed with changes.

1. An important criterion is not included in the comparison of simulators:

whether or not a simulator can generate data with varying cell numbers and sequencing depths.

I would like to clarify that some simulators regard cells as random observations, while other

simulators regard pairs of (cell, gene) as variables. For the latter simulators, such as

ZINB-WaVE, the simulated count matrix would have the same dimensions as the input real

count matrix; hence, how to do "repeated simulation (more cells)" (the authors' reply) is not as

obvious as it seems. I would suggest that the authors add a column in Table 1 about what are

regarded as variables and observations by each simulator.

We agree with this comment, but found it rather challenging to address this. For example, while

some simulators offer arguments to specify the desired number of simulated cells, they

internally resample from an empirical (e.g., library size) distribution to achieve this. Similarly, the

number of cells could be varied for some methods by passing a vector of the desired length by

manually re-sampling beforehand. We have now included an additional column, “# cells”, in

Table 1 trying to cover these cases: x for no, i.e., gene-cell pairs are truly linked (e.g.,

ZINB-WaVE); √ for yes, i.e., sampling from a fitted distribution (e.g., library sizes are modeled as

log-normal); (√) for yes via internal or manual re-sampling from empirical parameters.

4. Line 77. The summary of simulators in this paragraph is not very meaningful. Just because

certain simulators are not used for a specific benchmark study doesn't mean that they cannot be

used in the corresponding data analysis task. It could be just due to the choice of the author of

these benchmark studies, or simply that some simulators have not been published yet.

I still don't think the benchmark column in Table 1 provides useful information. The summary of

existing benchmark studies only provides limited information regarding the popularity of the

simulators and would favor older simulators, which are not necessarily better. In other words,

this column should be highly correlated with the publication years of simulators.

At least I would suggest the authors change the text to reflect this possible bias.

To decrease the emphasis on the frequency with which simulators are applied in benchmarks,

we have removed the column from Table 1 as suggested, and instead moved the corresponding

citations into the text (inside of the parentheses stating the number of benchmarks where a

given simulator has been applied, e.g., “method (3 [ref1, ref2, ref3])”). Furthermore, we agree

with the reviewer’s comments (usage != usability, publication date), and have added the

following statement in the text to reflect these:

“Yet, this listing of benchmarks and their use cases is not exhaustive; the frequency with

which simulators are applied in benchmarks need not speak for or against their

performance (e.g., long-lived and user-friendly methods might be favored); and, because

some simulators have not been applied to a given task does not mean they can not be.”

5. What is the rationale behind transforming the gene expression from count to the log-CPM

scale? It seems that many of the benchmark datasets used here are generated from the

droplet-based protocols, and people have argued that for these types of datasets, just using the

count values directly may be enough [1] [2]. In particular, library size normalization may

introduce unwanted artifacts [2]. Moreover, it seems that more than half of the considered

simulators use count-value-based models like NB or ZINB. Will using the log-CPM scale still be

suitable in this case? Comparison results should also be reported on the count scale.

The authors’ response is about the inapplicability of several criteria to raw counts. However,

even though linear scale may be inappropriate, simply using the log(1 + count) transformation,

without using library size normalization should be enough. This can reduce the issue of

heteroskedasticity without introducing additional artifacts. Moreover, the authors should

emphasize in the text that they used the same transformation for all simulators for a fair

comparison. I also suggest the authors add a discussion about the relative advantages and

disadvantages of using raw counts, CPM, log(1 + count), and log-CPM.

We disagree with the reviewer that it “should be enough” to compare summaries on the

log(1+count) scale. Unless all cells are sequenced to the same depth (and lack composition

effects), the count scale -- and therefore also, the log(1+count) scale -- gives values that are not

generally comparable. We accept that normalization can itself introduce artifacts, but these are

on a scale where expression values should be comparable; in addition, we are also talking here

about summaries that are compared between a real dataset and a simulated dataset, both of

which undergo the same set of normalizations.

To make these aspects clearer in the text, under Supplementary section “Quality control

summaries”, we have: i) specified explicitly in the table which input data is used for each

summary; and, ii) revised the paragraph preceding it as follows (amendments highlighted in

bold):

“Let X (Y/Z) denote the count (expression) matrix of a reference or simulated dataset

with genes G={g1

,...,gG} and cells C={c1

,...,cC} from batches b=1,...,B and clusters

k=1,...,K. Here, B is the number of batches, K is the number of clusters, Y and Z

correspond to log1p-transformed counts per million (CPM) and log-library size

normalized counts obtained with scater’s calculateCPM and logNormCounts,

respectively. For principal component-(PC-)based summaries, we ran scran’s

modelGeneVar (on Z) and getTopHVGs to select the n = 500 most highly variable

features, and scater’s calculatePCA to compute their first ncomponents = 50 PCs. The

same set of inputs specified below were used to compute the respective

summaries for all reference and simulated datasets.”

Reviewer #2:

Thank you for your responses to my comments, they were very clear and addressed my

questions. I think I now have a much better understanding of how you performed the evaluation

and your position of things such as the difference between "reference" and "de novo"

simulations. I would suggest that these could be incorporated into the paper to clarify these

points for future readers.

I still have some concerns about how the methods were used, particularly Splatter. From your

comments I believe that you have simulated clusters and batches using Splatter by generating

several independent datasets and concatenating them, rather than using the in-built functionality

for these scenarios.

Yes, we have refrained from using Splatter’s (and other methods’) in-built functionality for these

scenarios as they are de novo (i.e., reliant on user-inputs) and not capable of estimating

appropriate parameter settings from a reference.

On an additional note, we have attempted manually estimating parameters for multi-group

simulation with Splatter, and corresponded with the package author for advice; however, without

success. Using the author’s own words: “In terms of estimating these parameters [...] these are

fairly heuristic so that a reasonable estimation process won't necessarily produce a value that

would give the same effect in the simulation”. See our comment regarding batch simulation

below for an example.

This may have affected the metrics you have calculated and your evaluations as two splat

simulations are independent even if they use the same estimated parameters (i.e. Gene 1 in

one simulation has no correspondence to Gene 1 in another simulation).

We believe this is only partly true. Splatter samples gene means using rgamma with previously

estimated parameters, giving the impression that genes are not related between simulations.

However, R’s random number generation would give similar values for two rounds of sampling

if: i) the parameters passed to rgamma are similar; and, ii) a random seed has been set. Thus,

in principle, the (dis)similarity between genes would be retained across independent

simulations. Nevertheless, we agree with the reviewer’s subsequent comments and suggestion

#3: Including Splatter for type n comparisons only.

I think your motivation for doing this is to cover these scenarios without setting a user-defined

parameter; however, you still use the number of clusters/batches in the real dataset to decide

how many simulations to concatenate. Aside from the effect of this on the evaluation of Splatter,

I think it also disadvantages the other packages that can generate these scenarios with user

input but for which you haven't employed this same workaround (powsimR and scDesign).

In principle, all type n simulators could be used as type b and k with this workaround (i.e.,

concatenating independently simulated batches/clusters).

There are three suggested approaches I have for how to address this

(there could be other approaches you decide to try):

1. Keep the current approach but extend it to these other packages and clearly state in the text

how and why this has been done and that it is a non-standard way for these tools to be used

(not my preference but maybe the least amount of work).

We agree that this would be straight-forward to do and possibly “the least amount of work”.

However, the most consistent approach would then be to include all simulators in every

comparison (e.g., type n can be extracted from type b/k simulations, and type b/k can be

generated from concatenating type n simulations). As such, this workaround might be

misleading considering: i) the limited flexibility of available methods; and, ii) the seemingly bad

performance of methods not designed for the task under evaluation.

2. Use the inbuilt parameters for these scenarios for these packages. As I said, you are already

using these values from the real data so they could be passed directly to the packages rather

than using this workaround.

Perhaps there is a misunderstanding here. While parameters are estimated separately for each

batch/cluster from the real data, this can not be passed to Splatter for a multi-group simulation.

For example, simulating batches with Splatter requires specifying parameters of a log-normal

distribution (location and scale) from which scaling factors for each gene in every batch are

drawn. These are i) not obtained when estimating parameters separately for each batch; and; ii)

result in constant inter-batch differences (on average). As a result, although one could estimate

appropriate parameters for every batch pair, Splatter would take only one location-scale pair as

input.

This is just one example. Overall, we would emphasize again that we focused here on the

evaluation of reference-based simulators, and that it is not straightforward (and often not

possible) to mimic a real dataset with de novo simulation (e.g., by manually estimating

appropriate parameter settings).

3. Remove Splatter from the cluster and batch comparisons. This would be consistent with how

the other packages are currently treated and your decision to focus on reference-based

simulations.

In fact, we originally did not include Splatter for type b and k comparisons since the methods

reference-based simulation is limited to type n, and all other scenarios rely on additional

parameter inputs by the user. Thus, we agree that this (i.e., treating Splatter as type n only)

would be the most reasonable and consistent approach. Specifically, we have made the

following amendments:

- Removed types (,b/k) from scDesign, powsimR and splatter in Table 1 that previously

had a caption footnote stating “type b/k accommodated by joining type n simulations of

each group (batch/cluster).”

- Adjusted the stated number of type b/k methods accordingly: “We selected a total of 16

methods, 9/6 of which could accommodate batches/clusters (Tab. 1).”; previously we had

said 10/8 here.

- Revised the main text in various places to refrain from discussing Splatter in the context

of simulating batches/clusters and evaluating methods for integration/clustering.

- Updated all main and supplementary figures to reflect these changes.

It is possible that I have misunderstood your intentions or missed a technical reason why things

had to be done this way. If that is the case please correct me.

Whatever your approach, my main concern is that the packages are treated consistently and it

is clear to the reader when you have done something outside normal usage. This concern

extends to some of the current methods. While I understand your reluctance to expand the

methods section it should be clear what changes you have made to method usage without

having to search through the code. You state that "With few exceptions, methods were run using

default parameters and, if available, following recommendations given by the authors in the

corresponding software documentation" but there is no details of what these exceptions are. For

example, I noticed that in your script for powsimR you actually generate two populations and

select one to use for evaluation but that is not explained to the reader who may not look at the

code or have enough knowledge to interpret what it does or understand parameter names. I

don't expect you to explain the details of every method but please describe when you have

chosen to do something outside default usage. The supplementary methods you have for

datasets and metrics are excellent but this detail is missing for the simulation methods.

We have added an additional supplementary section “Method parameters” giving a description

of the (non-default) settings used for parameter estimation and data simulation.

**3^rd^ round**

**Reviewer 1**

Note that the main text of the R2 document was not compiled correctly, but I checked the authors' responses to my comments nevertheless.

The authors have addressed my comments 1 and 2, but not comment 3 about the use of log CPM versus other normalization procedures. I think they should at least explain the rationale for using log CPM in this study and mention there are other normalization procedures (e.g., the default Seurat is normalized by 10,000.) Another pitfall is that log CPM does not represent the raw data. For instance, if a simulator directly simulates CPM instead of raw counts, it may outperform other simulators that generate counts, but this does not mean that the log CPM simulator is better because its data does not reflect the variation in cell library sizes.

In short, my suggestion is that the authors may not need to run other normalization procedures, but the authors should discuss the above issues in the Discussion.

**Authors’ response**

Regarding Reviewer 1's comment, we must admit that we do not fully understand the comment, and I believe at least a variant of this comment has been present since the first version. We are very willing to discuss these points and modify the text, but we are not fully clear on what can be done. For now, we have added an additional comment in the Methods section when introducing the logCPM: "we chose logCPM as a summary statistic that is both a standard measure to compute, and comparable across reference and simulated datasets."
The Reviewer states "I think they should at least explain the rationale for using logCPM in this study and mention there are other normalization procedures (e.g., the default Seurat is normalized by 10,000.) Another pitfall is that logCPM does not represent the raw data. For instance, if a simulator directly simulates CPM instead of raw counts, it may outperform other simulators that generate counts, but this does not mean that the logCPM simulator is better because its data does not reflect the variation in cell library sizes." For one thing, we do not know of any simulator that simulates logCPMs; all of them simulate counts. But we have now discussed the rationale.
